# Cleavage of N-terminus of polycystin-1 increases calcium permeability of polycystin-1/2 receptor channel complexes

**DOI:** 10.1172/jci.insight.185186

**Published:** 2025-09-02

**Authors:** Runping Wang, Danish Idrees, Mohammad Amir, Biswajit Padhy, Jian Xie, Chou-Long Huang

**Affiliations:** Department of Internal Medicine, Division of Nephrology, University of Iowa Carver College of Medicine, Iowa City, Iowa, USA.

**Keywords:** Cell biology, Nephrology, Calcium channels, Calcium signaling

## Abstract

Mutations on genes encoding polycystin-1 (PC1) and PC2 cause autosomal-dominant polycystic kidney disease. How these 2 proteins work together to exert anticystogenesis remains elusive. PC1 resembles adhesion G-protein coupled receptors and undergoes autocleavage in the extracellular N-terminus to expose a hidden “stalk” region, which is hypothesized to act as a “tethered agonist.” Here, we show that WT PC1 and PC2 formed functional heteromeric channel complexes in *Xenopus* oocytes with different biophysical properties from PC2 homomeric channels. Deletion of PC1 N-terminus, which exposed the stalk, increased calcium permeability in PC1/PC2 heteromers that required the presence of stalk. Extracellular application of synthetic stalk peptide increased calcium permeation in stalkless PC1/PC2. Application of Wnt9B protein increased calcium permeability in PC1/PC2 but not in heteromers containing cleavage-resistant mutant PC1. Wnt9B interacted with N-terminal leucine-rich repeat (LRR) of PC1. Pretreatment with LRR blunted the increase in calcium permeability by Wnt9B. Thus, PC1 and PC2 form receptor-channel complexes that is activated by exposure of the stalk region following ligand binding to the PC1 N-terminus. The stalk peptide acts as a tethered agonist to activate PC1/PC2 by affecting ion selectivity of the complexes.

## Abstract 2

Mutations of polycystin-1 and 2 cause autodomal-diminant polycystic kidney disease. How the two prtoeins work together is unknonw. We show that they form receptor-channel complexes for external ligand to regulate cellular calcium signaling provide novel insights andpotential therapeutic strategy.

## Graphical abstract

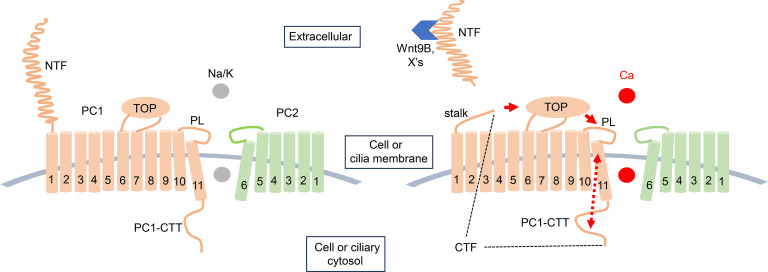

## Introduction

Autosomal dominant polycystic kidney disease (ADPKD) is a common life-threatening monogenic disease that leads to end-stage renal failure (1). This disease is characterized by development of fluid-filled cysts that compress the normal kidney tissue, resulting in structural and functional damage. About 50% of patients with ADPKD need dialysis or kidney transplantation by the fifth decade of life. The disease is predominantly caused by causal variants on *PKD1* and *PKD2* genes, which encode polycystin-1 (PC1) and PC2 proteins. Among these variants, ~80% are in *PKD1* and ~15% are in *PKD2* (2–4). ADPKD currently has no cure. Development of a new therapeutic strategy requires a better understanding of PC1 and PC2 function.

PC1 and PC2 partly function to regulate signals controlling cell growth and proliferation via intracellular calcium, cAMP, Wnt, and mTOR signals (5–7). Homozygous germline deletion of either gene in mice is embryonically lethal, whereas heterozygosity neither causes cysts nor death (8–10). A somatic second hit in heterozygous carriers, with gene dosage reduction of PC1 or PC2 below a threshold, is required for cystogenesis in humans (11–16). Changes in intracellular calcium concentration play an important role in the development of ADPKD (17, 18). Increases in intracellular calcium decrease cAMP level and mTOR activity, reduce cell proliferation, and increase autophagy (17, 19–24). Intracellular calcium concentration in primary culture of patients with ADPKD has been reported to be ~20 nM lower (17). Treatment with calcium ionophore or triptolide, which restores the calcium signal, arrests cell proliferation and attenuates cyst formation (17, 25).

PC2 protein has 6 transmembrane domains and intracellular N and C termini. It forms homotetramers and functions as a Ca^2+^-permeable, cation-selective channel. PC1 is a large protein (4,303 amino acids) consisting of a large extracellular N-terminus (3,074 amino acids), 11 transmembrane domains, and a short intracellular C-terminus of ~200 amino acid. The N-terminus contains multiple adhesion domains and a G-protein coupled receptor autoproteolysis inducing (GAIN) domain, resembling those found in adhesion G protein-coupled receptors (aGPCRs). PC1 (~520 kDa) is autocleaved at the GPCR proteolysis site (GPS) within the GAIN domain at the distal end of N-terminus adjacent to the first transmembrane domain (TM1). GPS cleavage produces a ~370 kDa N-terminal fragment (NTF) and a ~150 kDa C-terminal fragment (CTF). The CTF includes the 11 TM domains and a ~34 kDa cleavable intracellular C-terminal tail (CTT). N-terminus cleavage exposes a 21–amino acid peptide region immediately preceding the TM1 segment. This “stalk peptide” region, tethered to TM domain, is believed to function as a tethered agonist for PC1, much like the activation mechanism of aGPCRs (26, 27).

Structural studies show that four PC2 subunits form a homomeric channel characteristic of the TRP channels (28, 29). The last 6 TMs of PC1 resemble the TMs of PC2. The TM regions of PC1 and PC2, in the absence of intracellular termini, can assemble to form hetero-tetrameric channels (30). The cryo-EM structure of PC1/PC2 heteromers appears to represent a closed nonfunctional state. Whether the native heteromeric PC1/PC2 channel, consisting of WT full-length PC1 and PC2, is functional remains unclear. PCs are present in the ER, on the apical and basolateral membranes of kidney tubules, and in the primary cilium. In the primary cilium, PCs particulate in fluid shear stress–induced ciliary calcium response (31, 32). In ER, they regulate Ca^2+^ release through IP_3_ or ryanodine receptors (33, 34). PC2-null cells exhibit reduced agonist-induced ER Ca^2+^ release (35, 36). Conversely, exogenous expression of PC2 in HEK cells augment ER Ca^2+^ release (37). In mice or in proximal tubular cells, knockdown of PC2 and/or PC1 lowers the baseline Ca^2+^ level (18, 38, 39). Reexpression of full-length PC2 or PC1 in KO mice not only prevents disease progression but also reverses the pathology of ADPKD (40). Reexpression of the deleted genes reverses inflammation, extracellular matrix disposition, and fibrosis as well as kidney cysts.

Patch-clamp recordings of PC2 on the primary cilium reveal that PC2 preferentially conducts monovalent cations over Ca^2+^ (41). Gain of function of PC2 expressed in *Xenopus* oocytes exhibits similar permeability profile with higher K^+^ over Ca^2+^ permeability (42). We recently reported that PC2 functions as a K^+^-permeable channel that facilitates K^+^-Ca^2+^ exchange, enhancing ER Ca^2+^ release (43). Trimeric intracellular cation channel isoform-B (TricB), an ER-restricted K^+^-permeable channel, can complement PC2 deficiency in cells and mice. The findings that PC1 and PC2 physically associate and their TM domains coassemble raise the hypothesis that they form receptor-channel complexes (30, 41, 44). Studies of WT PC2 channel activity have so far been limited to patch-camp recording on cilia, lipid bilayers, and fluorescence Ca^2+^ imaging (33, 41, 45–49). In this study we demonstrated that WT PC2 can form functional channels in *Xenopus* oocytes. We then reconstituted PC1/PC2 heteromeric channels to examine the receptor-channel hypothesis. Our evidence suggests that PC1 N-terminus cleavage activates the complexes by increasing Ca^2+^ permeation through heteromeric channels. We confirmed that the secreted morphogen Wnt9B acts as a ligand that activates the complexes by releasing the autocleaved N-terminus. We compared our results with existing literature to further understand the functional and structural aspects of polycystins. Our results lay the foundation for future studies on structure-function relationships of the complex, the identification of additional ligands, and a further elucidation of regulatory mechanisms. Finally, our studies open the possibility for new therapeutic strategies targeting *PKD1* N-terminal mutations by focusing on downstream regions such as TOP domain.

## Results

### Expression of PC2 produces currents in Xenopus oocytes.

**Figure 1:**
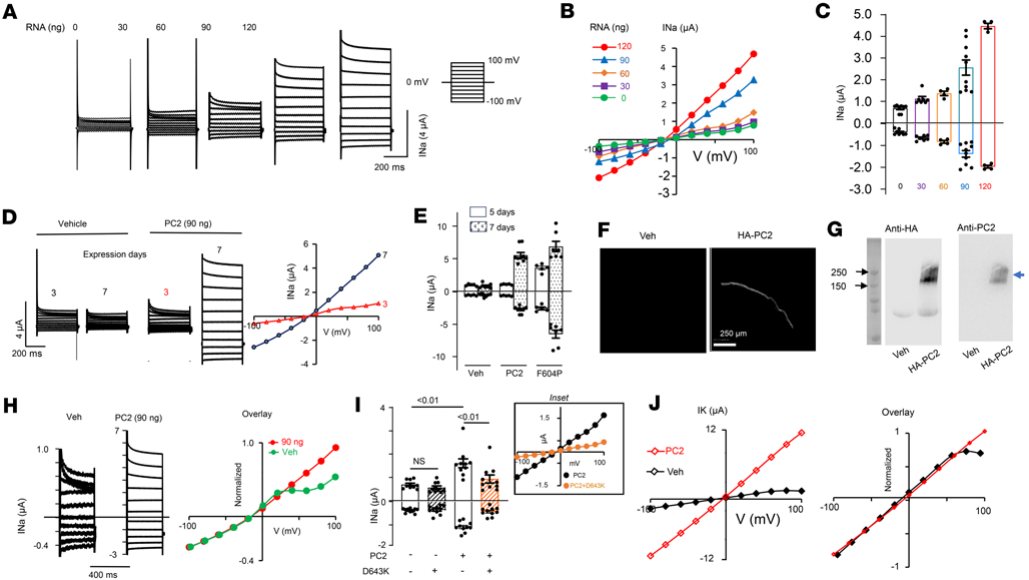
WT PC2 expresses currents in *Xenopus* oocytes. (**A**) Currents in *Xenopus* oocytes injected with vehicle (water), or mRNA coding for WT PC2. Currents were recorded by 2-electrode voltage-clamp in a bath solution with 100 mM NaCl. Holding potential was –50 mV, and 400 ms steps were applied from –100 mV to 100 mV in 20 mV increments. (**B**) Steady-state (at 400 ms) current-voltage (I-V) relationship curves from **A**. (**C**) Mean ± SEM for inward and outward currents at –100 and 100 mV. *n* = 8, 7, 4, 10, 4 for 30, 60, 90 and 120 ng mRNA injection, *P* <0.0001, 1-way ANOVA. (**D**) Step currents and steady-state I-V from oocytes recorded 3 or 7 days after injection. (**E**) Mean ± SEM for inward and outward currents from oocytes recorded at 5 or 7 days. *P* <0.01, 7 days versus 5 days for PC2 (90 ng) or F604P (30 ng mRNA injected); *P* < 0.001 for PC2 (*n* = 5) versus F604P (*n* = 5) at 5 days; *P* < 0.01 for PC2 (*n* = 9) versus F604P (*n* = 7) at 7 days. *n* = 5 and 7 (5- and 7-day) for vehicle injected group. (**F**) Immunofluorescence staining using antibody against HA tag. Scale bar: 150 μm. (**G**) Western blot of oocyte lysates probed by anti-PC2 or anti-HA antibody. Oocytes were injected with vehicle or C-terminal HA-tagged PC2 in **F** and **G**. Blue arrow: HA-tagged PC2; black arrow: 250 and 100 kDa protein standards. (**H**) Oocytes were injected with vehicle or PC2 mRNA. I-V curve of steady-state current at 400 ms were scaled and overlaid. (**I**) Oocytes were injected with vehicle or WT PC2 and with or without D643K PC2 mutant at 1:1 mRNA ratio. Inset showed I-V relationship, *n* = 10 for all. (**J**) Currents were recorded in a bath solution containing 100 mM KCl. Scaled and overlaid currents. Two-tailed unpaired Student’s *t* test for **E** and **I**.

PC2 channel activity has been measured on cell membrane, using reconstituted lipid bilayer recordings and by patch-clamp recordings of the primary cilium (33, 41, 45–47, 49–53). Yu’s group reported channel activity of gain-of-function (GOF) PC2 mutants in *Xenopus* oocytes (42, 54). We explored the possibility that WT PC2 currents could be detected in *Xenopus* oocytes with longer expression time (7 versus 2–3 days in most studies). Oocytes were injected with vehicle or 30–120 ng of mRNA coding for WT PC2 and recorded 7 days later by 2-electrode voltage clamp in a bath solution containing 100 mM NaCl. As shown, oocytes injected with ≥ 30 ng of mRNA expressed slightly outward-rectifying currents in a dose-dependent manner (Figure 1, A–C). The dose-dependent increase in currents occurred in both the inward and outward directions.

We further examined the time course of expression required to detect currents in oocytes injected with WT PC2 RNA. Currents were not detectable at 3–5 days after injection of 90 ng WT PC2 RNA but could be detected at day 7 (Figure 1, D and E). For comparison, in oocytes injected with 30 ng of GOF F604P-PC2 (phenylalanine-604 to proline) mutant mRNA, currents could be detected at day 5 (Figure 1E). We also injected 60 ng of F604P PC2 RNA, but the majority of cells died before recording at day 5. Cell death may be related to intracellular calcium overload and toxicity due to the higher current level of GOF mutant. In subsequent experiments, 90 ng of WT PC2 and 30 ng of F604P-PC2 RNA were used unless otherwise indicated. Expression of PC2 on oocyte membrane and in cell lysates was confirmed by immunofluorescence staining and Western blot analysis (Figure 1, F and G).

To examine whether currents observed in PC2-injected oocytes could be due to activation of endogenous channels, we compared the properties of currents recorded from vehicle- and PC2-injected oocytes. As shown in step-current tracings, the much smaller endogenous oocyte currents displayed time-dependent inactivation during depolarization, with current decay over the 400 ms holding period, such that steady-state current at 400 ms revealed an additional rectification at > 25 mV (Figure 1H). This was further evidenced by the steady-state current-voltage (I-V) relationship curves, where vehicle-injected currents were scaled up and overlaid with those from PC2-injected oocytes (right panel). While these results alone do not completely exclude that observed PC2 currents are not endogenous, the conclusion is further supported by experiments using a loss-of-function PC2 mutant, D643K-PC2 (aspartate 643 to lysine), as a dominant-negative inhibitor. Expressing D643K-PC2 had no effects on basal current in vehicle-injected oocytes, but it inhibited the coexpressed WT PC2 current (Figure 1I). PC2 channels recorded from the primary cilium exhibit lower Na^+^ over K^+^ permeability (41). Since K^+^ is the most abundant cation inside cell, this finding may contribute to slightly lower inward (mediated by Na^+^) than outward (presumably predominantly mediated by K^+^) current when recorded in a 100 mM NaCl bath. Consistent with the notion, replacing the bath with 100 mM KCl increased inward currents, giving a more linear I-V relationship in PC2-injected oocytes (Figure 1J). Differences between endogenous currents and PC2-mediated K^+^ currents were evident in overlaid, scaled-up I-V curves showing deviation at > 75 mV (Figure 1J). Together, these results support the notion that the currents recorded in PC2-expressing *Xenopus* oocytes are mediated by PC2 and not by the activation of endogenous channels.

### Coexpression with sPC1 increases PC2 surface expression and alters channel properties.

**Figure 2:**
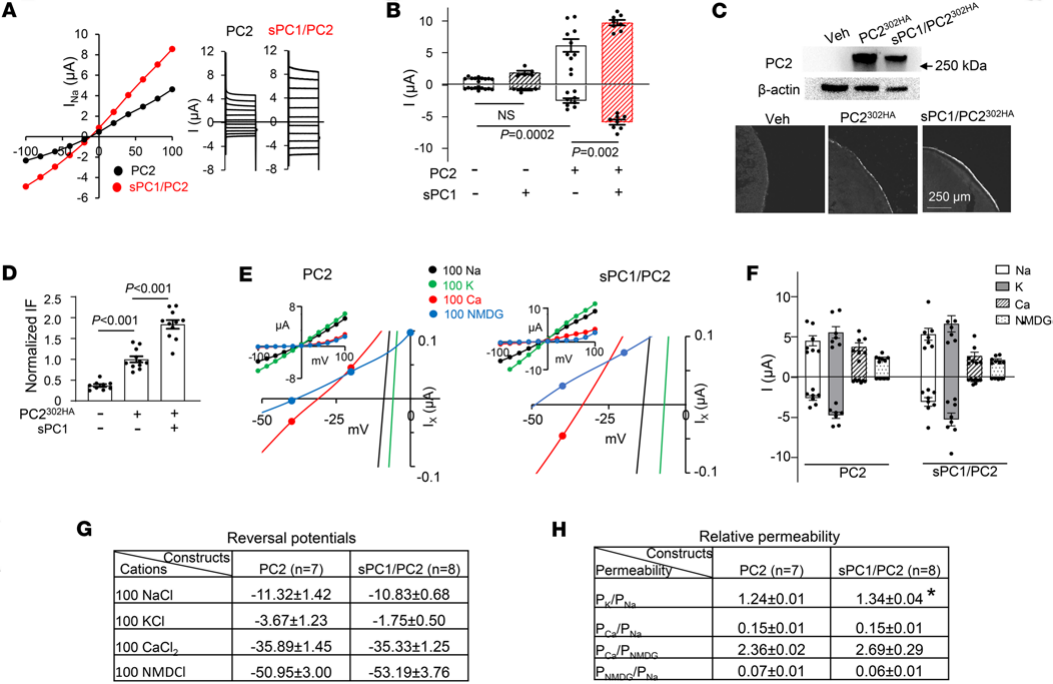
PC1 increases surface expression and K^+^ permeability of PC2. (**A**) I-V relationship of currents from oocytes expressing PC2 alone or sPC1/PC2 recorded in100 mM NaCl. (**B**) Mean ± SEM for inward and outward currents at –100 and 100 mV in oocytes injected with vehicle or mRNA for PC2, sPC1 or both; *n* = 10, 5, 8, and 8, respectively. (**C**) Oocytes injected with vehicle or mRNA for a HA-tagged PC2 (PC2^302HA^), with or without sPC1. PC2^302HA^ contains an extracellular HA tag engineered to replace amino acids 302–306 of the TOP domain (second extracellular loop) of PC2. Top: representative Western blot of total oocyte lysates probed with anti-PC2 and anti–β actin antibody. Bottom: representative immunofluorescence (IF) staining images using anti-HA antibody extracellularly in nonpermeabilized oocytes. IF staining in oocytes injected with sPC1 alone was not different from vehicle-injected (not shown). (**D**) Mean ± SEM of normalized IF intensity of surface expression of PC2^302HA^ in control (vehicle), PC2^302HA^, and sPC1/ PC2^302HA^-injected oocytes as in **C** (*n* = 9, 11, and 11, respectively). IF intensity was normalized to PC2^302HA^-injected oocytes. (**E**–**H**) Oocytes expressing PC2 or sPC1/PC2 were recorded in 100 mM NaCl, KCl, CaCl_2_, or NMDGCl. (**E**) Representative I-V with enlarged curves showing reversal potentials. (**F**) Mean ± SEM for inward and outward currents at –100 and 100 mV, *n* = 7 and 8 for PC2 and sPC1/PC2. (**G**) Reversal potentials. (**H**) Calculated relative permeability P_X_/P_Y_. *P < 0.01 for PC2 versus sPC1/PC2. Two-tailed unpaired Student’s *t* test for **B** and **D**–**F**. In all panels, experimental number (*n*) is number of oocytes as shown by scatter plots. All experiments were repeated 2 or more times with similar results.

Cryo-EM studies revealed that truncated PC1 and PC2 containing transmembrane domains form nonfunctional heteromers (30). Wang et al. show that PC1 coassembles with a GOF PC2 mutant to increase Ca^2+^ permeability (42). Ha et al. reported that in HEK cells, expression of a secretory PC1 (sPC1) carrying the κIgG signal sequence increases cell surface targeting of WT PC2, but functional PC2 currents could not be detected (48). Here, we tested whether WT PC1 and PC2 can form functional channel complexes on cell-surface membrane. To enhance cell-surface expression, we engineered a similar sPC1 construct with the κIgG leader sequence. Coexpression of sPC1 robustly increased the total current; the total current was ~2-fold larger in sPC1/PC2 coexpression compared with PC2 alone (inward currents 2.4-fold, outward current 1.6-fold; Figure 2, A and B). We examined surface expression using an extracellularly hemagglutinin-tagged (HA-tagged) PC2 construct (PC2^302HA^) and external application of anti-HA antibody without cell-membrane permeabilization. Coexpression with sPC1 increased surface expression of PC2 by ~1.86-fold (Figure 2, C and D; normalized to PC2 alone). The increased surface abundance of PC2 by sPC1 coexpression was not due to increased total protein abundance; in fact, the total PC2 in lysate from oocytes injected with sPC1/PC2 was slightly less than that from oocytes injected with PC2 (Figure 2C).

Next, we examined the relative permeability of Na^+^, K^+^, Ca^2+^, and NMDG^+^ (N-methyl-D-glucamine) for PC2 homomers and sPC1/PC2 heteromers by comparing reversal potentials in bath solution containing 100 mM NaCl, KCl, CaCl_2_ or NMDGCl. To minimize contamination of Cl^–^ current through calcium-activated Cl^–^ channel TMEM16A, experiments were performed in presence of the TMEM16A inhibitor T16A(inh)-A01 (10 μM). Figure 2E shows representative reversal potentials at 100 mM of each cation for PC2 homomers and sPC1/PC2 heteromers. Figure 2F shows mean inward and outward currents. As shown, the reversal potential for PC2 homomers in 100 mM NaCl, KCl, CaCl_2_, and NMDGCl were –11.32 ± 1.42, –3.67 ± 1.23, –35.89 ± 1.45, and –50.95 ± 3.0, respectively (Figure 2G). These results indicate order of permeability of K^+^ > Na^+^ > Ca^2+^ > NMDG^+^, consistent with previous reports for PC2 (41). Coexpression with sPC1 did not alter the sequence of permeability, nor did it change the reversal potential for Na^+^, Ca^2+^, and NMDG^+^, except for K^+^, where the reversal potential shifted by ~+2 mV. The relative permeability was estimated using changes in the reversal potential when 1 extracellular cation was replaced by equal molar of other cations (Figure 2H). These results conformed higher permeability for K^+^ over Na^+^, Na^+^ over Ca^2+^, and Ca^2+^ over NMDG^+^. As expected for the +2 mV positive shift in the reversal potential for sPC1/PC2 versus PC2 in 100 mM KCl, P_K_/P_Na_ for the former was slightly higher than the latter (Figure 2H). Thus, sPC1 coexpression alters the biophysical properties of PC1/PC2 heteromers, as evidenced by increased K^+^ permeation.

### GOF F604P-PC2 mutant is different from WT PC2.

**Figure 3:**
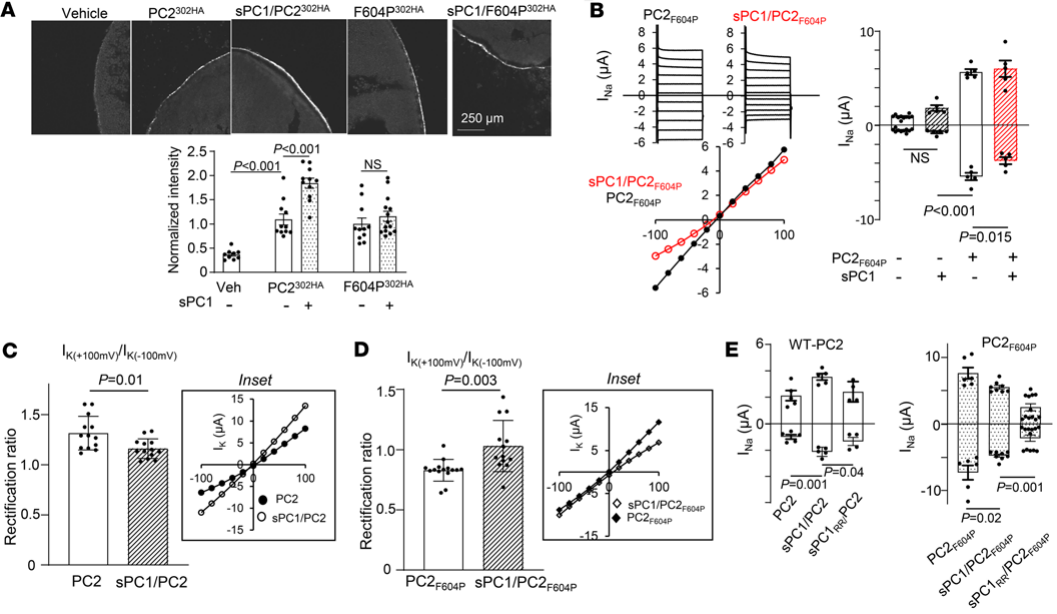
GOF mutation F604P-PC2 mutant is different from WT PC2. (**A**) Representative immunofluorescence (IF) images and mean ± SEM of normalized IF intensity of surface expression of PC2_F604P_ (labeled F604P^302HA^, *n* = 11) or F604P^302HA^/sPC1 (*n* = 14) as indicated. IF staining using anti-HA antibody against HA tag on the TOP domain as in Figure 2C. Note that the experiments in **A** were conducted concurrently with those shown in Figure 2, C and D; thus, the intensity of the IF images were comparable. Scale bar: 150 μm. (**B**) Representative current steps, steady-state I-V curve (at 400 ms), and mean ± SEM for inward and outward currents at –100 and 100 mV in oocytes injected with mRNA for F604P-PC2 (PC2_F604P_) or PC2_F604P_/sPC1 as indicated. *n* = 5 for each group. Experiments were conducted concurrently with those shown in Figure 2B; refer to Figure 2B for the values of the vehicle and sPC1-injected groups. (**C**) Rectification ratio of PC2 and sPC1/PC2 currents recorded in 100 mM KCl bath. The ratio of outward K^+^ current (at +100 mV) to inward K^+^ current (at –100 mV) is shown. *n* = 13 each group. Inset: I-V curve. (**D**) Rectification ratio for PC2_F604P_ and sPC1/PC2_F604P_ as illustrated in **C**. *n* = 13 each group, inset: I-V curve. (**E**) Effect of sPC1 carrying R4100E and R4107E mutation (sPC1_RR_) on inward and outward currents of heteromeric channels formed with PC2 and PC2_F604P_. *n* = 4–5 each group. Two-tailed unpaired Student’s *t* test for **A**–**E**. In all panels, experimental number (*n*) is number of oocytes as shown by scatter plots. All experiments were repeated 2 or more times with similar results.

Because homomeric WT PC2 channel currents could not be recorded on the cell membrane, studies on PC2 channels have relied on using GOF mutants (42, 48, 54). One previous study reported that PC1 coexpression decreases F604P-PC2 currents in *Xenopus* oocytes (54), while another in HEK cells showed that PC1 increases the expression of F604P (48). Here, we compared the effect of sPC1 coexpression on WT PC2 and F604P-PC2 mutant. In contrast to its effect on WT PC2 (Figure 2, C and D), we found that sPC1 coexpression had no effect on cell-surface expression of F604P-PC2 (Figure 3A). sPC1 coexpression decreased inward current but had no effect on outward current of F604P, resulting in more outward rectification for sPC1/F604P-PC2 heteromers (Figure 3B). The finding that outward current was not altered is consistent with the observation that cell surface expression was unchanged. The shift in rectification indicates that sPC1 and F6404P-PC2 coassembly forms heteromers with different biophysical properties compared with sPC1/WT-PC2. The amino acid phenylalanine-604 resides on the fifth TM segment of PC2. Mutation of phenylalanine to proline produces a kink in the TM segment, leading to a constitutive open pore (54). We reason that this structural alteration in F604 mutation affects subunit assembly with sPC1, affecting cell surface expression and biophysical properties of sPC1/F604P-PC2 compared with sPC1/WT-PC2. To further support that coassembly of F604P versus WT-PC2 with sPC1 underlines the differences in biophysical properties of heteromers, we examined the rectification ratio (outward current at +100 mV over inward current at –100 mV). The rectification ratio for WT-PC2 and F604P homomers were different (Figure 3, C and D). Moreover, sPC1 coexpression decreased the rectification ratio for heteromers formed with WT-PC2, while increased it for those formed with F604P. Thus, GOF PC2 mutants including F604P should not be used as functional readout of increased PC2 channel activity in vivo, despite exhibiting increased channel activity. The cryo-EM structure of PC1/PC2 heteromers reveals that 3 positively charged amino acids, arginine-4100 (R4100), arginine-4107 (R4107), and histidine-4111 (H4111) on the eleventh TM segment of PC1, protrude side chains into the ion conducting pathway (30). Supporting the role of these residues in ion conduction, charge neutralization and size-chain reduction of these residues by mutating them to glycine increases conductance of heteromeric channel formed with a GOF PC2 (42). We examined the effect of charge conversion of these residues by mutating them to glutamate. Coexpression of sPC1 carrying R4100E and R4107E mutations (sPC1_RR_) with PC2 decreased currents of heteromeric channels (Figure 3E). The same effect of sPC1_RR_ was observed on F604P-PC2. These results support the notion that PC1 is a pore forming subunit in heteromeric PC1/PC2 channels.

### Cleavage of N-terminus of PC1 increases Ca^2+^ permeability of heteromeric sPC1/PC2 channel.

**Figure 4:**
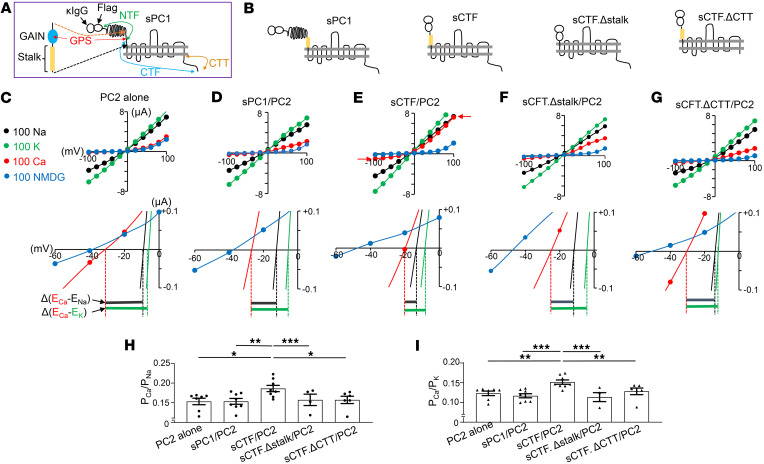
Removal of N-terminus of PC1 at GPS site increases Ca^2+^ permeability of the heterotetramer. (**A**) Schematic illustration of the sPC1. κIgG: κ IgG light chain signal peptide; Flag, Flag antibody epitope tag; NTF, N-terminal fragment; CTF, C-terminal fragment; CTT, intracellular C-terminal tail. Cleavage at GPS site (G-protein coupled-receptor domain proteolytic site) results in NTF and CTF. NTF contains multiple adhesion domains for protein and carbohydrate interaction and a partial GAIN domain sequence before GPS. CTF contains stalk peptide region, 11 transmembrane domains, and the C-terminal tail (CTT). The enlarged region shows GAIN domain (GPCR auto-proteolysis inducing, blue) that contains GPS and stalk peptide (yellow). Full-length PC1 is 4,303 amino acids; NTF is 3,048 amino acids; CTF is 1,255 amino acids; CTT is ~186 amino acids. Not drawn to scale. (**B**) Scheme of sPC1 deletion constructions used. (**C**–**G**) Representative I-V curves for PC2 alone or with different sPC1 deletion constructs recorded in bath solution containing (in mM) 100 NaCl (black), 100 KCl (green), 100 CaCl_2_ (red), or 100 NMDG-Cl (blue). Selected regions of I-V curves are enlarged to show reversal potentials. Reversal potential shifts from CaCl_2_ to NaCl [labeled Δ(E_Ca_-E_Na_)] and to KCl [Δ(E_Ca_-E_K_)] are shown. In **E**, red arrows indicate that Ca^2+^ entry through heteromers leads to increased inward current size and show that Ca^2+^-activated Cl^–^ current contributes to increases in outward current size (**E** versus other panels). TMEM16A channel inhibitor, T16A(inh)-A01 (10 μM), does not cause complete inhibition of endogenous Ca^2+^-activated Cl^–^ currents. (**H** and **I**) The relative permeability of Ca^2+^ versus Na^+^ (P_Ca_/P_Na_) and Ca^2+^ versus K^+^ (P_Ca_/P_K_) were calculated from potential shifts upon switching extracellular solution. Individual *n* numbers for each group are shown in Supplemental Table 1. *P < 0.001, **P < 0.002, ***P < 0.01, by 2-tailed unpaired Student’s *t* test.

Autocleavage of the N-terminus of PC1 exposes a hidden stalk region, which activates PC1 signaling via a tethered-agonist mechanism (26, 27). Having established that PC1 and PC2 form functional receptor-channel complexes, we hypothesize that stalk peptide plays a key role in activating these complexes. To test the hypothesis, we generated a series of PC1 constructs that replicate the effect of NTF cleavage, deletion of the exposed stalk region after cleavage, and/or deletion of the intracellular C-terminus (CTT) (Figure 4, A and B). The construct sCTF includes the κIgG signal sequence and the CTF, which contains the stalk region. The construct sCTF.Δstalk is sCTF lacking the stalk peptide. The construct sCTF.ΔCTT has the CTT deleted from sCTF.

As described in experiments shown in Figure 2, E–H, we measured the reversal potential for each cation at 100 mM concentration in the bath, and we calculated relative permeability based on the shift in reversal potential (Figure 4, C–I, and Supplemental Table 1; supplemental material available online with this article; https://doi.org/10.1172/jci.insight.185186DS1). Figure 4, C–G, show representative I-V curves illustrating reversal potentials for Na^+^, K^+^, and Ca^2+^ and reversal potential shifts at 100 mM CaCl_2_ relative to 100 mM NaCl [labeled Δ(E_Ca_-E_Na_)] and to 100 mM KCl [Δ(E_Ca_-E_K_)]. The calculated relative permeability ratios (P_Ca_/P_Na_ and P_Ca_/P_K_) based on these reversal potential shifts are shown (Figure 4, H and I, and Supplemental Table 1). As already shown in Figure 2, E–H, coexpression of WT sPC1 did not affect the Ca^2+^ permeability of sPC1/PC2 heteromers (Figure 4, C, D, H, and I; sPC1/PC2 versus PC2 alone). Of note, the reversal potential for K^+^ shifted slightly toward more positive relative to Na^+^ (Figure 2E and Figure 4, C and D), reflecting an increase in P_K_/P_Na_ in sPC1/PC2 heteromers versus PC2 alone (Supplemental Table 1). Importantly, coexpression of sCTF with PC2 caused a positive shift in reversal potential for Ca^2+^ (E_Ca_) such that Δ(E_Ca_-E_Na_) and Δ(E_Ca_-E_K_) were smaller for sCTF/PC2 than for sPC1/PC2 (Figure 4, D and E), and calculated relative permeability, P_Ca_/P_Na_ and P_Ca_/P_K_, were increased (Figure 4, H and I). Thus, NTF cleavage in PC1 increases Ca^2+^ permeability in PC1/PC2 heteromers.

To test the hypothesis that the increase in Ca^2+^ permeability is due to the exposure of stalk peptide acting as a tethered agonist, we examined the effect of sCTF.Δstalk. We found that Ca^2+^ permeability in sCTF.Δstalk/PC2 was reduced compared with sCTF/PC2 (Figure 4, F, H, and I), supporting the essential role of the stalk peptide in activating the channel. Deletion of the intracellular C-terminus of PC1 (CTT) abolished the increase in Ca^2+^ permeability caused by NTF cleavage (Figure 4, E and G–I), indicates that CTT is necessary for the increase in Ca^2+^ permeability following NTF cleavage. Supporting that NTF cleavage in PC1 alters ion permeability/selectivity in PC1/PC2 heteromers, we found that the sCTF/PC2 heteromers have reduced permeability to tetraethylammonium (TEA) (Supplemental Figure 1). For comparison, PC2 homomers and sPC1/PC2 heteromers have significantly higher permeability to TEA.

### External application of the stalk peptide increases Ca^2+^ permeability in stalkless N-terminus–cleaved heteromeric channel.

**Figure 5:**
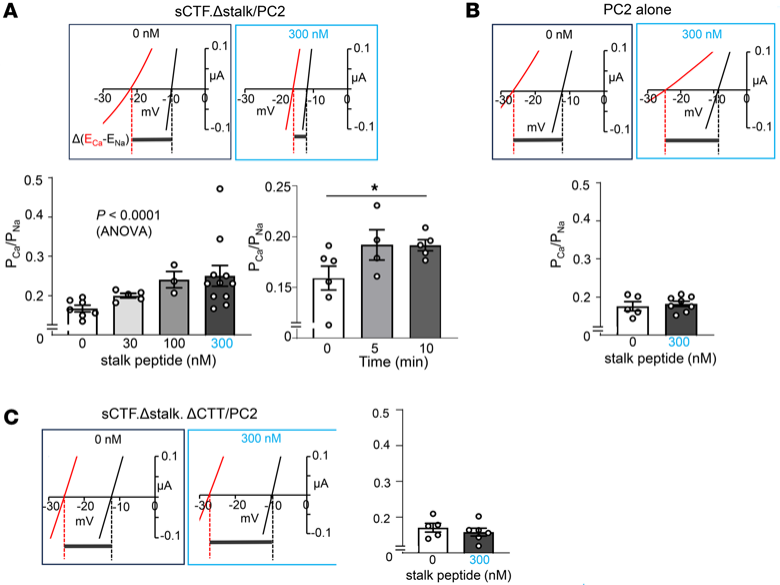
External application of stalk peptide increases Ca^2+^ permeability of heteromeric channel formed by PC2 and stalkless sCTF. (**A**) Top: oocytes expressing sCTF.Δstalk/PC2 were incubated with synthetic stalk peptide at 0, 30, 100, or 300 nM for 4 hours. The 21–amino acid peptide corresponds to residues 3049-TAFGASLFVPPSHVRFVFPEP-3069 of human PC1. Bottom left: P_Ca_/P_Na_ was calculated based on reversal potential shift between 100 mM CaCl_2_ and 100 mM NaCl. Data are shown as mean ± SEM. *P* < 0.0001 by 1-way ANOVA.; *n* = 7, 5, 3, and 12 for 0, 30, 100, and 300 nM, respectively, of stalk. Bottom right: time course of stalk peptide (300 nM) on increases in P_Ca_/P_Na_. (**B** and **C**) No effect of stalk peptide (300 nM) on PC2 alone (*n* = 5 and 8 for 0 and 300 nM peptide) (**B**) or sCTF.Δstalk.ΔCTT/PC2 (*n* = 5 and 6 for 0 and 300 nM peptide) (**C**). Two-tailed unpaired Student’s *t* test for **B** and **C**. In all panels, experimental number (*n*) is number of oocytes as shown by scatter plots. All experiments were repeated 2 or more times with similar results.

To further support the hypothesis that the stalk peptide is important for activating PC1/PC2 heteromers, we examined the effect of synthetic stalk peptides on Ca^2+^ permeability in sCTF.Δstalk/PC2. Oocytes expressing the heteromers were incubated in a solution containing 0, 30, 100, or 300 nM of stalk peptide for 4 hours and tested for relative Ca^2+^ versus Na^+^ permeability. Compared with control without peptide, treatment with stalk peptide ranging from 30 to 300 nM caused dose-dependent increases in Ca^2+^ permeability in heteromers containing stalkless N-terminus–cleaved PC1 (Figure 5A). In separate experiment, we showed that effect of stalk peptide to increase Ca^2+^ permeability occurred within 10 minutes after application. The effect of stalk peptide is mediated by PC1, as the maximal concentration of stalk peptide (300 nM) had no effect on oocytes expressing PC2 alone (Figure 5B) nor on heteromers consisting of stalkless CTF with an additional CTT deletion (Figure 5C). The requirement for CTT in increasing Ca^2+^ permeability by stalk peptide was demonstrated in Figure 4, G–I.

### Wnt9B plays a role in increasing Ca^2+^ permeability, which requires PC1 N-terminus cleavage.

After autocatalytic cleavage at GPS site, NTF remains attached to CTF noncovalently (55). Release of the noncovalently attached NTF is important for complete biological function of PC1 (48, 56, 57). Wnt9B has been shown to interact with PC1 NTF (58). Here, we tested the hypothesis that Wnt9B is involved in the release of NTF, thereby increasing the Ca^2+^ permeability of PC1/PC2 heteromers. Wnt9B is a secreted protein that undergoes posttranslational lipidation and tends to aggregate in the absence of natural binding proteins or carriers (59, 60). We expressed Wnt9B in serum albumin-containing media (with albumin acting as a carrier) and purified in the detergent CHAPS (3-[(3-Cholamidopropyl)dimethylammonio]-1-propanesulfonate hydrate) (Supplemental Figure 2). The activity of the purified Wnt9B was validated by its ability to activate the canonical Wnt signaling pathway in HEK293T cells. HEK293T cells possess an intact Wnt/β-catenin signaling pathway that responses to canonical Wnt ligands by preventing β-catenin degradation, leading to its accumulation. Western blot analysis showed that HEK293T cells treated with Wnt9B for 16 hours resulted in elevated β-catenin levels (Supplemental Figure 3).

**Figure 6:**
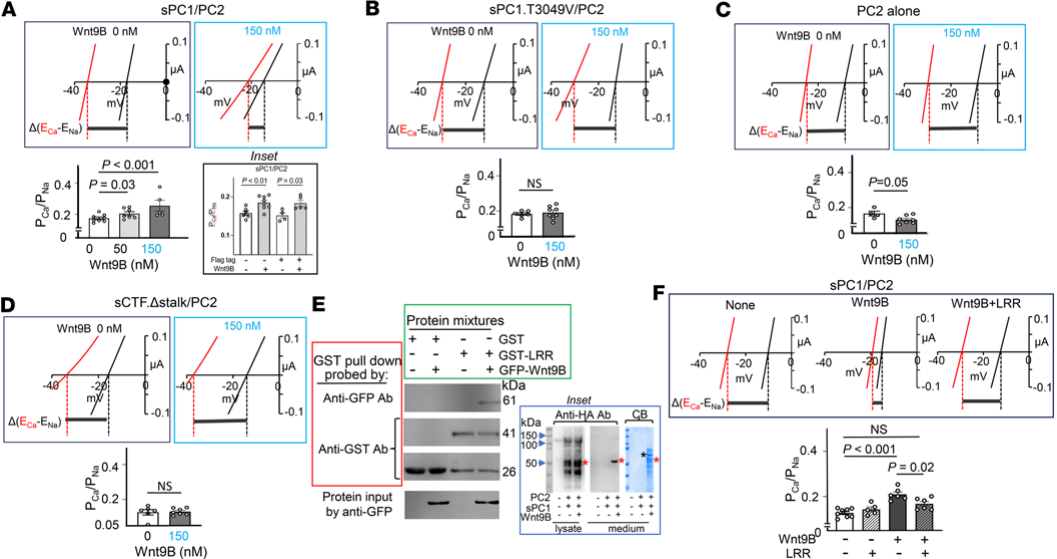
Purified Wnt9B protein increases Ca^2+^ permeability of heteromeric sPC1/PC2 channel. (**A**–**D**) Oocytes expressing sPC1/PC2 (*n* = 8, 7, and 5 for 0, 50, and 150 nM Wnt9B, respectively) (**A**), sPC1.T3049V/PC2 (*n* = 6 and 8 for 0 and 150 nM Wnt9B, respectively) (**B**), PC2 alone (*n* = 4 and 7 for 0 and 150 nM Wnt9B, respectively) (**C**), or sCFT.Δstalk/PC2 (*n* = 6 and 7 for 0 and 150 nM Wnt9B, respectively) (**D**) were incubated overnight with either vehicle or purified Wnt9B (50 or 150 nM). P_Ca_/P_Na_ was calculated based on reversal potential shift between 100 mM CaCl_2_ and 100 mM NaCl. Inset in **A** shows that sPC1 with or without Flag-tag (plus PC2) responded to Wnt9B. (**E**) Biochemical interaction between PC1 leucine-rich repeat (LRR) and Wnt9B. Mixtures containing purified GFP-Wnt9B, GST-LRR, or GST alone were subjected to pull-down using GST-sepharose beads. Pull-down samples were probed with anti-GFP or anti-GST antibodies. Protein input samples were probed by anti-GFP Ab. Note that purified GST-LRR fusion proteins also contained some cleaved GST alone. Inset shows a ~51 kDa HA-tagged NTF fragment is released into the medium in the presence of Wnt9B. Control or oocytes expressing sPC1/PC2 were incubated with or without Wnt9B (300 nM) overnight. sPC1 contains an N-terminal HA-tag immediately after the κIgG signal peptide. The medium was concentrated 10-fold for Western blot analysis by anti-HA antibody. Proteins in the medium were also stained by Coomassie blue (CB). Red and black asterisks indicate HA-tagged NTF fragment and GST-Wnt9B, respectively. Experiment was repeated 3 times. (**F**) Oocytes expressing sPC1/PC2 were incubated overnight with vehicle or purified Wnt9B (150 nM), with or without LRR (1,500 nM). P_Ca_/P_Na_ was determined as in **A**–**D**. Data are shown as mean ± SEM. *n* = 8, 5, 6, and 6 for no treatment, LRR, Wnt9B, and LRR+Wnt9B treated groups, respectively. Two-tailed unpaired Student’s *t* test for **A**–**D** and **F**.

When oocytes expressing sPC1/PC2 heteromers were incubated with Wnt9B at 50 or 150 nM, there was an increase in Ca^2+^ permeability compared with controls treated with vehicle buffer (Figure 6A). The N-terminal flag-tag in the sPC1 does not contribute to the effect by Wnt9B, as removing the tag did not alter the results (Figure 6A). Wnt9B had no effects on oocytes coexpressing PC2 and a NTF cleavage-resistant mutant PC1.T3049V (threonine-3049 to valine) (Figure 6B), supporting the notion that NTF cleavage is required. Wnt9B had no effects on oocytes expressing PC2 alone (Figure 6C), indicating the increases in Ca^2+^ permeability is dependent on PC1. Finally, Wnt9B did not increase Ca^2+^ permeability in oocytes coexpressing stalkless CTF with PC2 (Figure 6D), indicating that the effect requires NTF and is not mediated by interactions with other regions of PC1, such as pore loop. To confirm that Wnt9B is a PC1 ligand, we showed that it interacted with PC1 leucine-rich repeat (LRR) in pull-down assays (Figure 6E). In addition, incubation with purified LRR fusion protein blocked the ability of Wnt9B to increase Ca^2+^ permeability (Figure 6F), further supporting the role of Wnt9B-LRR binding in the process. Supporting the hypothesis that Wnt9B promotes the release of cleaved NTF, incubating oocytes expressing sPC1/PC2 with Wnt9B resulted in the appearance of a ~51 kDa protein band in the culture medium (Figure 6F). The effect is dose-dependent, with 300 nM Wnt9B treatment causing significantly more release of the ~51 kDa NTF fragment compared with 100 nM (Supplemental Figure 4A). Release of this fragment occurred as early as 0.5 hours after incubation with 300 nM Wnt9B. The abundance of the NTF fragment was less after 16 hours compared with 0.5 hours after incubation, suggesting that degradation of the fragment likely occurs in the medium over the time period.

In parallel, we also examined changes in Ca²^+^ permeability of the heteromeric channel complex following Wnt9B treatment for 0.5 and 16 hours. Despite a detectable release of NTF fragment in the medium, we found that Ca²^+^ permeability was not increased after 0.5 hour treatment with Wnt9B (Supplemental Figure 4B). A significant increase in P_Ca_/P_Na_ ratio was evident after 16 hours of treatment. Immediately after NTF release, the stalk peptide may not be exposed in the correct position to function as a tethered agonist. Alternatively, the stalk peptide may need to involve other regions or adopt certain conformational changes. These questions will be investigated in future studies. Western blotting by anti-HA antibody and peptide sequencing revealed that the fragment represents a smaller fragment of released NTF that contains the N-terminal HA tag, LRR, and CTL domains of PC1 (Figure 6E and Supplemental Figure 5).

### Ca^2+^ permeation through PC1/PC2 heteromeric channels at physiological Ca^2+^ concentration.

**Figure 7:**
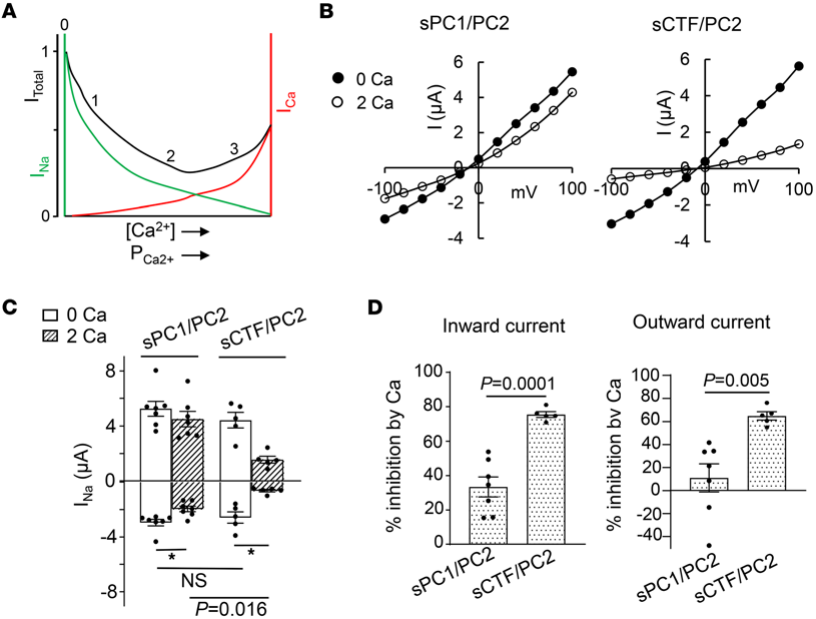
PC1 N-terminal cleavage enhances extracellular Ca^2+^ inhibition of Na^+^ current through heteromeric sPC1/PC2 channels. (**A**) Illustration of anomalous mole fraction effect. Total current (black trace, I_Total_) is the sum of Na^+^ current (green, I_Na_) and Ca^2+^ current (red, I_Ca_). (**B**) Representative I-V curve of currents in oocytes expressing sPC1/PC2 or sCTF/PC2 in 100 mM NaCl bath with 0 or 2 mM external Ca^2+^. (**C**) Mean ± SEM of inward and outward currents at –100 and 100 mV for experiments shown in **B**. *n* = 5–8 for each group as shown by scattered plots. *P < 0.05. (**D**) Percentage inhibition of inward and outward currents by 2 mM external Ca^2+^ from experiments in **C**. Two-tailed unpaired Student’s *t* test for **C** and **D**. All experiments were repeated 2 or more times with similar results.

Experiments using 100 mM extracellular Ca^2+^ demonstrated that NTF cleavage increases Ca^2+^ permeability in PC1/PC2 heteromers (Figure 4, Figure 5, Figure 6). Here, we examined the hypothesis at the physiological extracellular Ca^2+^ levels. The low surface/cytosol ratio in large cells like oocytes poses a technical challenge for using fluorescence Ca^2+^ imaging as a tool to detect small Ca^2+^ influx. The existence of other conductive pathways and the lack of specific inhibitor for PC1/PC2 confound the reliability of reversal potential shift measurement induced by the small 2 mM physiological [Ca^2+^]. A common feature of Ca^2+^-permeable channels is the inhibition of monovalent cation current by extracellular Ca^2+^ (61), which is known as anomalous mole fraction effect (AME). This effect occurs because Ca^2+^ permeation through the pore blocks the passage of other monovalent cations. Thus, when the magnitude of Ca^2+^ currents carried by permeating Ca^2+^ ions is smaller than the reduction in monovalent cation currents, the total currents decrease in the presence of Ca^2+^. As illustrated in Figure 7A, at zero or low Ca^2+^ concentration, Na^+^ ions (I_Na_) permeate through Ca^2+^-permeable channels (point 0). With increased Ca^2+^ permeation through the pore (shown as I_Ca_), either through higher external Ca^2+^ concentrations or enhanced Ca^2+^ permeability, Na^+^ current is reduced, such that the total current (I_total_) decreases (point “1” and “2”). As Ca^2+^ concentration or permeability increase further, total current I_total_ increases when I_Ca_ exceeds ΔI_Na_ (point “3”).

As expected from the low Ca^2+^ to Na^+^ permeability ratio and the principle of AME, physiological extracellular Ca^2+^ concentration inhibits Na^+^ current through PC2 (41). We utilized the AME property to examine Ca^2+^ permeation in PC1/PC2 heteromers in the physiological Ca^2+^ concentration. As shown, addition of 2 mM Ca^2+^ to a bath containing 100 mM Na^+^ inhibited the currents in PC1/PC2 heteromers (Figure 7, B and C). Consistent with our notion that NTF cleavage increases Ca^2+^ permeation through heteromers (Figure 7A), addition of 2 mM Ca^2+^ caused a bigger inhibition of current in sCTF/PC2 (Figure 7, B and C). The percentage inhibition of inward as well as outward current were both increased for sCFT/PC2 than for sPC1/PC2 (Figure 7D), suggesting that Ca^2+^ permeation through the pore blocks Na^+^ permeation in both directions. Of note, although the macroscopic Ca^2+^ currents (I_Ca_) through sCTF/PC2 at 2 mM external Ca^2+^ were too small to be detected by 2-electrode voltage clamp here, these currents were evident at 100 mM Ca^2+^ (Figure 4E).

### Ca^2+^-mediated inhibition of monovalent cation current is not due to intracellular Ca^2+^-mediated inactivation.

**Figure 8:**
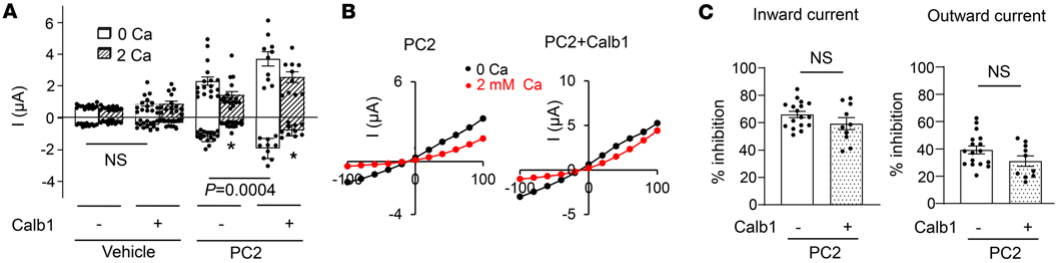
Effect of calbindin-1 (Calb1) on PC2 current and external Ca^2+^-induced current inhibition. (**A**) Currents in oocytes injected with vehicle or PC2 mRNA, with or without Calb1. Currents were recorded in 100 mM NaCl bath with 0 or 2 mM external Ca^2+^. Mean ± SEM of inward and outward currents at –100 and 100 mV are shown. *n* = 11–17 for each group as shown by scattered plots. (**B**) Representative I-V curves for experiments in **A**. (**C**) Percentage inhibition of inward and outward currents by 2 mM external Ca^2+^ in PC2 with or without Calb1 from experiments in **A**. Two-tailed unpaired Student’s *t* test for **A** and **C**. All experiments were repeated 2 or more times with similar results.

Ca^2+^-dependent inactivation occurs in some Ca^2+^-permeable channels, serving to limit rises in intracellular [Ca^2+^] and prevent Ca^2+^ overload (62, 63). The effect of intracellular Ca^2+^ on PC2 Na^+^ currents has been reported to be multiphasic, with a bell-shaped relationship. Increasing intracellular Ca^2+^ ([Ca^2+^]_i_) up to ~10 μM increases PC2 activity positive-cooperatively, but higher concentration result in inhibition (37, 41). To support that inhibition of Na^+^ current by 2 mM Ca^2+^ is due to AME rather than intracellular Ca²^+^ feedback, it is important to assess whether Ca^2+^-dependent inactivation contributes to current inhibition by external Ca^2+^ under our experimental setting. We first tested it by expressing calbindin-1 (Calb1), a calcium-binding protein, to buffer the intracellular Ca^2+^. Expression of Calb1 had no effect on endogenous current in vehicle-injected oocytes but increased both inward and outward currents in oocytes expressing PC2 (Figure 8A). Importantly, Calb1 did not affect the inhibition of PC2-mediated current caused by extracellular Ca^2+^ (Figure 8, B and C), suggesting that intracellular Ca^2+^ feedback does not contribute to the inhibition.

**Figure 9:**
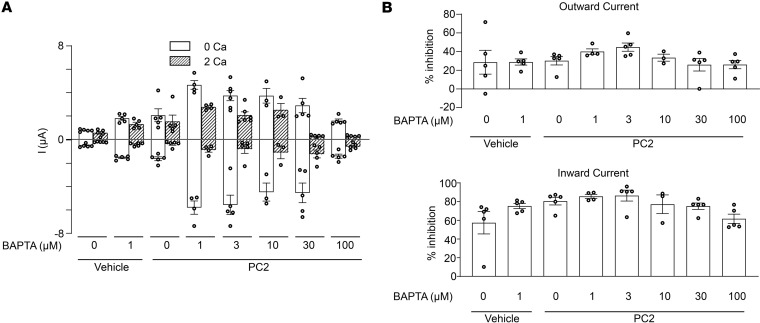
Effect of BAPTA-AM on PC2 current and external Ca^2+^-induced current inhibition. (**A**) Oocytes injected with vehicle or PC2 mRNA were incubated with BAPTA-AM at the indicated concentration for 2 hours before recording currents in 100 mM NaCl bath solution with 0 or 2 mM external Ca^2+^. Mean ± SEM of inward and outward currents at –100 and 100 mV are shown. *n* = 3–5 for each group as shown by scattered plots. *P* = 0.0002 by 1-way ANOVA. (**B**) Percentage inhibition of inward and outward currents by 2 mM external Ca^2+^ in vehicle or PC2-injected oocytes at indicated BAPTA-AM concentration. *P* = 0.09 by 1-way ANOVA for both, not statistically different for inhibition of either inward or outward current. All experiments were repeated 2 or more times with similar results.

To ensure that lack of effect by Calb1 is not due to slow Ca^2+^ buffering, we tested the effect of a kinetically fast-acting calcium chelator BAPTA-AM (64). Pretreatment of PC2 expressing oocytes with 1–30 μM BAPTA-AM (for 2 hours) resulted in increase in the basal current (i.e., 0 extracellular Ca^2+^), while pretreatment with higher concentration of BAPTA-AM at 100 μM led to a decrease in current (Figure 9A). Importantly, as with Calb1, presence of fast buffer BAPTA-AM did not affect external Ca^2+^-mediated inhibition of current (Figure 9, A and B), further supporting that extracellular Ca^2+^-induced inhibition of Na^+^ current through PC2 is not due to increases in local intracellular Ca^2+^, which exert feedback inhibition on the channel. Our results also confirm previous reports of biphasic regulation on PC2 by intracellular Ca^2+^, although it does not contribute to the external Ca^2+^ inhibition under our experimental setting.

## Discussion

*PKD1* and *PKD2* genes were identified about 3 decades ago. The functions of PC1 and PC2 and how they exert anticystogenic effects remain unclear. Progress has been partially hampered by the lack of simple heterologous expression system to reconstitute polycystin channel function. So far, the channel function of WT PC2 has been studied by patch-clamp recordings on cell membrane, primary cilium, and on reconstituted lipid bilayer recordings (33, 41, 45–47, 49–53). Several studies have examined GOF mutant PC2 in oocytes (42, 54). While channel activity of WT PC2 was difficult to be detected by electrophysiology, Wang et al. observed calcium uptake in oocytes coexpressing WT PC1 and PC2 using calcium-45 isotope (42). In the present study, we unequivocally demonstrate that WT PC2 exhibits functional currents in *Xenopus* oocytes when expressed for a longer time course than is typically used in most studies. The PC2 currents in oocytes, recorded by 2-electrode voltage clamp, share fundamentally comparable properties with those recorded on the primary cilium. Importantly, WT and GOF PC2 differ in their biophysical properties and regulation, raising questions about the reliability of GOF PC2 as a readout for activation of PC2 activity in vivo.

Biochemical and structural studies suggest that PC1 and PC2 may form heteromeric complexes. The ability to express functional WT PC2 currents in oocytes allows us to demonstrate that they form complexes with functionalities. Coexpression with PC1 increases PC1/PC2 heteromeric channel currents by increasing PC2 cell surface expression. Ha et al. also observed increase in PC2 surface expression in HEK cells with PC1 coexpression but did not detect functional currents (48). PC1 and PC2 physically interact through a coiled-coil interaction at the intracellular C-terminus (65). More recent cryo-EM studies reveal that the truncated transmembrane domains of PC1 and PC2 form presumably nonfunctional hetero-tetramers in 1:3 stoichiometry (30). In the present study, we find that PC1/PC2 form heteromers with different biophysical properties from PC2 homomers, evident by an increase in the relative K^+^ to Na^+^ permeability *(*P_K_/P_Na_) and a decrease in the outward rectification. Moreover, mutations in PC1 putative pore-lining residues convert PC1 into a dominant-negative subunit. These results support the notion that both PC1 and PC2 contribute to forming the pore.

**Figure 10:**
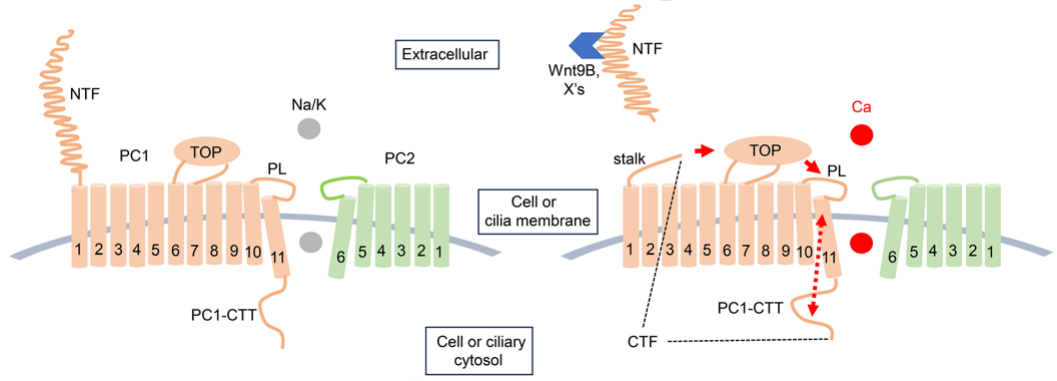
Working model for Wnt9B action of heteromeric PC1/PC2 channel. PC1 contains 11 transmembrane (TM) domains, while PC2 has 6. The last 6 TMs of PC1, together with PC2, form heteromeric channels. Autocleavage of PC1 at GPS site produces NTF and CTF (right panel; the region CTF is marked by dotted lines). The cleaved NTF remains noncovalently attached to the heteromeric CTF/PC2 channels. The complexes exist on cell or ciliary membrane. Normally, PC1/PC2 heteromers preferentially conduct monovalent cations K^+^ and Na^+^ over Ca^2+^. Secreted Wnt9B and other unidentified potential ligands (labeled X) bind to the complex extracellularly facilitating the release of NTF. This process exposes the hidden stalk peptide, which act as a tethered agonist to increase the Ca^2+^ permeability of heteromers. How stalk peptide does this remains unknown, it may involve interaction with the TOP domain, which in turn interacts with the pore loop (PL) of PC1 (depicted by red arrows), as suggested recently by Pawnikar et al. (26, 27). Notably, the C-terminal tail of PC1 (PC1-CTT), located immediately downstream of the pore, likely influence ion permeation (depicted by arrowed red broken line). Only the TOP domain of PC1 is illustrated in the model. Shear stress may promote detachment of the noncovalently bound NTF under certain conditions (48), though this is not sufficient by itself in our experimental setting, where oocytes were subject to continuous shaking on a platform shaker for 7 days after mRNA injection.

PC1 forms heteromers with PC2 in ER soon after protein synthesis. PC1 undergoes N-terminus cleavage at GPS sites to generate NTF and CTF fragments (Figure 10). Formation of heteromers is important for NTF cleavage (57, 66, 67). The cleaved NTF remains noncovalently attached to CTF/PC2 complexes. NTF cleavage is important for the exit of PC1/PC2 from ER-Golgi en route to their destined membrane sites, including the primary cilium and the cell membrane (57, 68). PC1 shares structural features with adhesion GPCRs. Like adhesion GPCRs, the stalk region exposed after autocatalysis and the release of NTF is postulated to act as a tethered agonist to activate PC1-CTF (44). Recently, Pawnikar et al. provided support for this hypothesis (26, 27). They showed that HEK cells expressing engineered stalkless PC1 alone exhibited reduced NFAT-luciferase reporter activity, which could be stimulated by synthetic stalk peptide applied extracellularly. Computational modeling by the authors suggested that tethered stalk-mediated activation may involve stalk-TOP (tetragonal opening for polycystins) domain-pore loop interaction to increase PC1-CTF signaling. A previous study showed that a 222 amino acid intracellular CTT of PC1 activates the NFAT reporter (26). However, whether stalk-dependent activation of the NFAT reporter observed by Pawnikar et al. involves the 222 amino acid CTT has not been tested. In the present study, we show that PC1 and PC2 form functional heteromeric channels and that the tethered stalk activates the heteromers. Interestingly, the activation requires the presence of the last 186 amino acids in the C-terminus. Furthermore, we confirm that Wnt9B is a ligand that activates the heteromers. Thus, PC1 and PC2 form bona fide receptor-channel complexes. An early study reported that Wnt9B interacts with LRR domain of PC1 and increases Ca^2+^ influx in HEK cells expressing PC1/PC2 (58). In the present study, we show that the PC1 stalk region is both necessary and sufficient to increase Ca^2+^ permeability in heteromers. Autocleavage and tethering of cleaved NTF to CTF/PC2 in oocytes expressing PC1 and PC2 have been demonstrated by Wang et al. (42). Here, we provide direct evidence that Wnt9B interaction with the NTF promotes its release and increases Ca^2+^ permeability in the PC1/PC2 heteromers. Thus, Wnt9B, by promoting the release of the tethered cleaved NTF, exposes the stalk region to function as a tethered agonist. Whether the stalk peptide acts through interaction with the TOP domain and the pore loop as proposed by Pawnikar et al. remains to be investigated. We have attempted to investigate whether the stalk domain-TOP interaction as reported by Pawnikar et al. (26, 27) may be involved in the increases in Ca^2+^ permeability we observed. Pawnikar et al. reported that electrostatic interactions between Asp-3072 in the stalk domain and Arg-3891 in the TOP domain and between Arg-3848 in the TOP and Glu-4078 in the pore loop are important for stalk-TOP-pore loop interaction to regulate PC1-mediated intracellular signaling (26, 27). They found that charge neutralization of respective residues decreases the signaling. In contrast, we observed that mutations of these residues increased Ca^2+^ permeability (Supplemental Table 2). There are 2 important factors to consider when comparing ours and their results. First, point mutation itself may alter protein structure beyond the intended effect of charge neutralization. Second, the study by Pawnikar et al. (26, 27) focuses on PC1-mediated intracellular signaling, whereas our studies investigate PC1/PC2 heteromultimers.

Wnt9B is a secreted protein that regulates kidney tubule morphogenesis (69), which overlaps with some aspects of PC1 function in development (70). The cellular signaling of Wnt proteins is mediated through canonical Wnt/β-catenin signaling pathway, the noncanonical planar cell polarity pathway, or via intracellular Ca^2+^ signaling (71). Our study suggests that 1 action of Wnt9B in tubular morphogenesis may involve the release of tethered cleaved NTF from CTF/PC2 complexes to activate a Ca^2+^ signaling cascade. The overwhelming abundance of PC2 over PC1 suggests that polycystins exist in at least 2 different populations in the ER: PC1/PC2 heteromers and PC2 homomers. Our findings suggest that these 2 populations may have different roles. For example, PC2 is important for PC1-CTT cleavage (72, 73), and mitochondria translocation of cleaved CTT plays a significant role in PC1 function (74). Ca^2+^ permeation through ER-localized PC1/PC2 may elevate local cytosolic [Ca^2+^] around the heteromers to regulate the cleavage and translocation of CTT through the ER-mitochondria contact. The N-terminus of PC1 contains multiple domains for protein and carbohydrate interactions. NTF-attached CTF/PC2 complexes are present in the primary cilium, on apical and basolateral membrane, as well as in the ER. Carbohydrates and protein components present in urine, cell-cell, and cell-basement membrane junctions may serve as external ligands that activate PC1/PC2 receptor-channel complexes. One such example is the basement membrane component laminin-511, which interacts with PC1-LRR (75). Additionally, shear stress from urinary flow or tension across cell to cell, or cell to matrix, may also contribute to the activation.

Structural studies of PC2 reveal 2 narrow regions within ion permeation pathway that serve as gates (76). One is the selectivity filter, defined by amino acids 641-LGD-643 (leucine-glycine-aspartate), which is stabilized by a preceding pore helix-1 (PH1) and a following pore helix-2 (PH2). Together, the PH1-selectivity filter-PH2 region forms the pore loop that connects the fifth (S5) and sixth (S6) transmembrane segment. Leucine-677 and arginine-681, located on the more distal part of S6 segment, protrude their side chains into the ion permeation pathway, serving as a lower gate. Wang et al. reported that mutating both leucine-677 and arginine-681 resides to alanine (LNAA) results in a constitutive-active GOF PC2 channel by widening the lower gate (42). Like WT PC2 homomers, LNAA-PC2 homomers are more selective for K^+^ than Ca^2+^. Wang et al. further showed that formation of PC1 and LNAA-PC2 heteromeric channels increases Ca^2+^ permeability and that N-terminus cleavage is not necessary for this increases. Our present results using WT PC1 and PC2 show different results. We show that formation of WT PC1/PC2 heteromeric channels does not, by itself, lead to increased Ca^2+^ permeability. Rather, NTF cleavage is necessary to increase Ca^2+^ permeability in PC1/PC2 heteromers. The discrepancy between our results and report by Wang et al. (42) likely reflects the 2 different PC2 constructs (WT versus GOF-PC2) used, respectively. Based on our results and the literature, we propose a working model for activation of receptor-channel complexes (Figure 10). In this model, PC1 N-terminal ligands bind and facilitate NTF release to expose the stalk peptide to act as a tethered agonist on PC1 TOP domain. This, in turn, interacts with the pore loop of PC1 to activate PC1/PC2 heteromers. LNAA mutation in PC2 induces conformational changes in the selectivity filter of tetramers in a manner recapitulated by the effect of the stalk peptide. The intracellular C-terminus of PC1 affects the selectivity filter through the S6 pore helix.

PC2 channels recorded on primary cilia exhibit a permeability order of K^+^ > Na^+^ > Ca^2+^ (41). A similar order has been reported for GOF PC2 expressed in oocytes (42, 54). We observed the same sequence of order for WT PC2 in our study. The relative permeability of K^+^ over Ca^2+^ (P_K_/P_Ca_) between these studies are slightly different. P_K_/P_Ca_ recorded on primary cilia has been reported as 10:1 to 40:1 (41, 49). The P_K_/P_Ca_ in our study is ~8:1. The cryo-EM structure reveals that TM domains of PC1 and PC2, without intracellular termini, coassemble to form asymmetric heteromers in a 1:3 stoichiometry (30). In this structure, PC1 lacks the typical pore loop composed of PH1-selectivity filter-PH2, as seen in PC2. Instead, the N-terminal end of eleventh TM segment, which is equivalent to S6 of PC2, called S6a, forms the selectivity filter with PH1. The pore region of heterotetrameric channels consists of one S6a from PC1 and three PH1s from PC2 subunits. Given this asymmetry, it is interesting to note that the relative permeability ratio between PC2 homomeric channels and PC1/PC2 heteromeric channels in our study does not differ dramatically. We found only a slight increase in P_K_/P_Na_ for PC1/PC2 heteromers compared with PC2 homomers (1.34 ±0.04 versus 1.24 ±0.01; Figure 2H). Importantly, NTF cleavage leads to an increase in P_Ca_/P_K_ and P_Ca_/P_Na_.

Our current results indicate that full-length PC1 and PC2 with intact intracellular termini can form functional channels. Moreover, deleting the last 186 amino acids (aa 4,118–43,03) of the PC1 C-terminus prevents the stalk peptide from increasing Ca^2+^ permeability in sCTF/PC2 heteromers. These results support the notion that the intracellular C-terminus of PC1 is necessary to maintain the correct structure of the conducting pathway and influence ion permeation through the pore. The NTF also affects the pore structure. PC2 homomers and sPC1/PC2 heteromers have significant permeability to the large inorganic molecule TEA (molecular size, ~130 kDa). Heteromers consisting of NTF-cleaved sCTF and PC2 have reduced TEA permeability. The overall structural implications of these results remain unclear but support the notion that NTF cleavage affects the pore structure of sPC1/PC2 heteromeric channels, directly or indirectly through conformational changes.

In conclusion, we show that PC1 and PC2 form functional receptor-channel complexes and confirm that Wnt9B is an activator ligand for the complexes. Mechanistically, external ligands activate the complexes by facilitating the release of cleaved, tethered N-terminus of PC1, thereby exposing the stalk domain to activate heteromeric channel. The activation leads to increased Ca^2+^ permeation, which can result in global or local increases in intracellular Ca^2+^ level. Future studies will include structure-function of the complexes, identification of additional ligands, and further elucidation of the regulatory mechanism. Our results provide proof of principle for therapeutic strategies targeting *PKD1* N-terminal mutations by direct targeting of downstream regions, such as TOP domain. Finally, it should be cautioned that our experiments were performed on heterologous expression system. The implication of our results in vivo and in diseased states such as ADPKD remains to be investigated.

## Methods

Supplemental Methods are available online with this article.

### Sex as a biological variant.

Female *Xenopus* were used for harvesting oocytes for expression of PC1 and/or PC2 channels.

### Statistics.

Data are presented as mean ± SEM. Experimental *n* number (number of oocytes) is illustrated by scatter plot. Statistical comparisons between two groups of data were performed using 2-tailed unpaired Student’s *t* test. Multiple comparisons were done using 1-way ANOVA followed by Šidák’s or Tukey’s multiple comparison tests. Electrophysiological data were analyzed using Clampfit 10.7 (Molecular Devices). *P* values of less than 0.05 were considered significant. Statistical analyses used in each experiment were also indicated in figure legends.

### Study approval.

All experimental procedures were conducted in accordance with the *Guide for the Care and Use of Laboratory Animals* (National Academies Press, 2011) and were approved by the IACUC at the University of Iowa Carver College of Medicine.

### Data availability.

All data associated with this study are available in the Supporting Data Values file.

## Author contributions

RW, DI, MA, and BP designed the study, conducted the experiments, analyzed the data, and participated in writing the paper. JX and CLH supervised the project. RW wrote the initial draft and CLH wrote the final paper. All authors approved the final version of the submitted manuscript.

## Supplementary Material

Supplemental data

Unedited blot and gel images

Supporting data values

## Version 1 09/02/2025

In-Press Preview

## Version 2 10/08/2025

Electronic publication
