# Supplementary material for: Cleavage of N-terminus of polycystin-1 increases calcium permeability of polycystin-1/2 receptor channel complexes
Source: JCI Insight. 2025 Sep 2;10(19):e185186. doi: 10.1172/jci.insight.185186 (PMC12513491; doi:10.1172/jci.insight.185186)
Supplement: Supplemental data [file jciinsight-10-185186-s221.pdf]

Supplementary information for:

Cleavage of N-terminus of polycystin-1 increases calcium permeability of polycystin-1/2 receptor channel complexes

Runping Wang, Danish Idrees, Mohammad Amir, Biswajit Padhy, Jian Xie,

and Chou-Long Huang

Department of Internal Medicine, Division of Nephrology, University of Iowa Carver College of Medicine, Iowa City, Iowa, USA

The supplementary file contains detailed methods, 5 supplementary figures, 5 supplementary tables, and un-cut get for Figure 1G, 2C and 6E.

Methods

Antibody, reagents, and preparation. Rabbit polyclonal anti-HA antibody (Abcam, ab9110), rabbit polyclonal anti-beta actin antibody (ThermoFisher Scientific, PA1-16889), mouse anti-PC2 monoclonal antibody (Santa Cruz, SC-28331), goat anti-mouse IgG-HRP (Invitrogen, 31430), goat anti-rabbit IgG-HRP (Southern Biotech, 4030-05), T16Ainh-A01 (Sigma-Aldrich, SML0493), and BAPTA-AM (Invitrogen, B1205) were used in this study. T16Ainh-A01 was dissolved in DMSO. Stalk peptide (synthesized by Genscrip) was dissolved in DMSO to make a 4 mM stock solution.

cDNA constructs and RNA synthesis. Sequence corresponding to human *PKD2* was cloned into pcDNA3.1 vector with an HA tag inserted at the C-terminus. For the PC2^{302HA} construct, amino acids 302-306 of *PKD2* were replaced with the HA tag. Full-length human *PKD1* cDNA was cloned into the pcDNA3.1 vector. The sPC1 constructs was created as described by Liu et al (1), with the endogenous 23 amino acids signal peptide at the N-terminus of PC1 replaced by an Ig kappa (*k*)-chain leader sequence, followed by a FLAG tag. Three copies of HA tag were added to the C-terminus of the sPC1, except the construct used for western blot and immunohistochemistry in Figure 2C and Figure 3A. The CTF construct included human PC1 amino acids T3049-T4303, the CTF.Δstalk spanned amino acids T3070-T4303, the CTF.ΔCTT spanned T3049-G4117. All constructs included the Ig *k*-chain leader sequence at N-terminus, followed by a FLAG tag, and contained 3 copies of HA tag at the C-terminus. The full length Wnt9B cDNA was cloned into the mammalian expression vector pEG BacMam (Addgene, 160680) with a His8 GFP tag attached to the N-terminus of Wnt9B. For the GST-LRR fusion protein construct, amino acids 24-176 of PC1 was subcloned into a pGEX-4T-1 vector. Mutagenesis was generated by PCR using Q5® Site-Directed Mutagenesis Kit (New England Biolabs, E0554S) or QuickChange II XL Site-Directed Mutagenesis kit (Agilent Technologies, 200521-S). All mutations were confirmed by sequencing. mRNAs were synthesized by in vitro transcription using Hiscrib T7 ARCA mRNA kit (with tailing, from NEB, E2060S).

Xenopus oocytes preparation and mRNA injection. *Xenopus* laevis (Xenopus I, cat# 4800) were housed in a static water tank at 16-18 °C with a 12-hour dark/light cycle. To harvest oocytes, female frogs were anesthetized by bathing in 0.15% Tricaine. After abdominal incision, 2-3 lobes of ovaries removed. The oocytes clumps were treated with 1.5 mg/ml collagenase type-4 and 1mg/ml trypsin inhibitor (Worthington,

LS003087) in OR2 solution (82 mM NaCl, 2 mM KCl, 1 mM $MgCl_2$, and 5 mM HEPES, titrated to pH of 7.4 using NaOH) at room temperature for 120-180 minutes. After digestion, the oocytes were incubated overnight in ND-96 (96 mM NaCl, 2 mM KCl, 1 mM $MgCl_2$, 1.8 mM $CaCl_2$, 5 mM HEPES, 2.5 mM sodium pyruvate, and 100 mg/l geneticin) at 18°C. For injection, mRNAs (90–120 ng) were diluted in 100~150 nl RNase-free water. Oocytes were incubated in ND-96 solution at 16-18°C for 7 days, with continuously shaken on a rotating platform shaker.

Electrophysiology. Seven days after mRNA injection, whole-cell currents were recorded by two-electrode voltage-clamp at room temperature (~25 °C) using an Oocyte Clamp amplifier (OC-725C, Warner Instrument Corp), Digidata-1322A digitizer, and Pclamp 9.0 software (Axon Instrument, Union City, CA, USA). Pipettes (resistance 1-2MΩ) were filled with 3mM KCl. Recording bath solution contained 100 mM NaCl, 5 mM HEPES, 2 mM Na-EGTA, pH 7.4 titrated with Tris-base. To test the reversal potential for different ions, bath solution was modified to contain 100 mM *X*Cl, 5 mM HEPES, and 2 mM EGTA, where *X* was Na^+, K^+, Ca^{2+} TEA^+ or $NMDG^+$. Voltage protocol held the potential at -50 mV, stepping from -100 mV to 100 mV in 20 mV increments, with each step last for 400 ms (at 1 sec interval). The recording chamber was grounded using agar bridge containing 3 mM KCl and junction potential was nulled. Relative permeability was calculated according to the equation (1):

$$\frac{P_x}{P_{Na}} = \frac{\alpha_{Nae}}{\alpha_{xe}}\left[\exp\left(\frac{E_{rev}}{RT/F}\right)\right]$$

where E_{rev}, α, R, T and F are reversal potential, effective activity coefficient for cations ("e" for extracellular), universal gas constant, absolute temperature, and Faraday constant, respectively. Effective activities (α_x) were calculated as: $\alpha_x = \gamma_x[x]$ where [x] is the concentration of ions and γ_x is the activity coefficient with values of 0.79, 0.72, 0.30 and 0.24 for Na^+, K^+, Ca^{2+} and $NMDG^+$, respectively (1, 2). Liquid junction potentials were subtracted from the reversal potentials. The liquid junction potentials were measured by first filling the bath solution with 100 NaCl. The offset was zeroed, and the bath solution was then switched to 100 mM KCl, $CaCl_2$, TEA-Cl and NMDG-Cl. The resulting voltage reading corresponds to the liquid junction potential with opposite polarity. The values obtained were within the range in literature (3) (-5 to +12 mV). The junction potential for 100 mM $CaCl_2$, KCl, TEA-Cl and NMDG-Cl were -7.07, +3.87, -3.17 and -5.57 mV respectively. The oocytes

were recorded the same day for each experiment. In each group of data obtained, we tried to obtain similar number of recordings for different DNA construct or condition.

In all figures, experimental number (*n*) is number of oocytes. All experiments were repeated two or more times with similar results.

Western blot analysis. Oocytes were rinsed 3 times in PBS and then frozen in -80°C overnight. After thawing on ice, 5 oocytes were homogenized in 0.5 ml of lysis buffer containing 50 mM HEPES, 0.5 M NaCl, 5% glycerol, 0.1% Triton X-100 with proteinase inhibitor (cOmplete mini 1 tablet/10 ml from Roche, REF 11836170001). After centrifuge for 10 minutes, the supernatants were mixed with NuPAGE LDS sample buffer containing 10% 2-Mercaptoethanol, heated to 95°C for 10 minutes, and then loaded onto NuPAGE 4-12% Bis-Tris gel. After separated by electrophoresis, proteins were transferred to PVDF Immobilon-P transfer membrane. After blocking with blocking buffer for 1 hour at room temperature, the PVDF membranes were incubated overnight at 4 °C in primary antibody with 1:2500 dilution in blocking buffer. The membrane was washed with TBST (Bio Rad, 1706435 with 0.1% tween-20) and then incubated for 1 hour at room temperature with secondary antibody conjugated with horseradish peroxidase in 1:10000 dilution. After 4 washes with TBST, the membranes were incubated with clarity western ECL substrate (Bio Rad, 170-5060) and imaged using an chemiluminescent imaging system. The density of the protein bands was analyzed using Image J software.

Immunohistochemical staining of surface membrane PC2. Xenopus oocytes expressing $PC2^{302HA}$ or $F604P^{302HA}$ with and without sPC1 (does not contain HA tag) were harvested 7 days after mRNA injection. $PC2^{302HA}$ and $F604P^{302HA}$ contains an extracellular HA tag engineered to replace amino acids 302-306 of the TOP domain (2nd extracellular loop) of PC2. Oocytes were fixed in 4% paraformaldehyde (ThermoScienfic, J19943-K2) overnight at 4°C. After washing with PBS, oocytes were blocked with 5% goat serum in PBS for 2 hours at room temperature and then incubated with primary antibody with a 1:2000 dilution in blocking buffer for 2 days. After washing with PBS, the oocytes were then incubated with goat anti–rabbit AlexaFluor-568 (Invitrogen, catalog A21069) with 1:500 dilution in blocking buffer for 2 hours at room temperature. The oocytes were then incubated sequentially in 10, 20, 30% sucrose overnight at each sucrose concentration at 4°C to

dehydrate. Oocytes were embedded in Scigen Tissue-Plus O.C.T compound (Fisher Healthcare, 23-730-571), frozen in -80°C, and sectioned (10 μm) using a microtome. After freezing in -20°C overnight, O.C.T compound was removed, and sections were mounted. Pictures were taken using Zeiss LSM 710 inverted confocal microscopy. Pictures were pseudo-colored. For the staining of C-terminus-HA-tagged PC2, oocytes were sliced first before blocking and staining with antibodies.

Expression and purification of Wnt9B protein. HEK293S cells expressing Wnt9B (pEGBacMam-Wnt9B) were cultured in DMEM medium with 5% fetal bovine serum. Purification of secreted Wnt9B protein was performed with slight modification from previous protocol (4, 5). Briefly, culture media at 72 hours post-transfection were passed through the Ni-NTA column, followed by washing with TBS (20 mM Tris, 150 mM NaCl, pH 7.5) containing 20 mM of imidazole to remove non-specifically bound material. The bound proteins were then eluted with 300 mM imidazole in TBS. The eluates were incubated overnight with TBS with 1% CHAPS, and further purified by size exclusion chromatography Superdex (GE Healthcare). Peaked fractions containing Wnt9B-serum albumin complexes were dialyzed against a buffer containing 20 mM Tris pH 7.5 and 1% CHAPS to reduce the ionic strength, subjected to 1-ml HiTrap Q-Anion column (Amersham Biosciences), and eluted stepwise in 1% CHAPS TBS with the increasing concentration of NaCl.

Expression and purification of LRR protein. E. coli BL21 DE3 cells were transformed with PGEX-*4T-1* containing PC1-LRR. The IPTG-induced bacterial culture was centrifuged at 4000 rpm for 10 min at 4°C. The pellets were dissolved in lysis buffer (50 mM Tris–HCl buffer, pH 7.5, 400 mM NaCl, 10 % (v/v) glycerol, 5 mM BME, DNase, 0.1 mg/ml Lysozyme, protease inhibitor cocktail, 2 mM phenyl methane sulfonyl fluoride (PMSF), 0.1 % (v/v) Triton X-100 (U.S. Biochemical Corp.), and 0.1% Tween-20). Cell lysis was performed using sonication on ice for 4 minutes and centrifuged at 13,000 rpm for 30 minutes. The supernatants were loaded to a glutathione affinity column equilibrated with buffer (50 mM Tris–HCl buffer, pH 7.5, 400 mM NaCl, 10 % (v/v) glycerol, 5 mM BME). The GST-LRR fusion proteins were eluted with elution buffer (50 mM Tris–HCl buffer, pH 7.5, 400 mM NaCl, 10 % (v/v) glycerol, 5 mM BME, 5-20 mM glutathione).

Wnt9B-LRR pull-down assays. Purified GST-tagged LRR or GST proteins (30 μg) in 500 μl assay buffer (20mM Tris pH 7.5, 400mM NaCl and 10% Glycerol) were incubated with the 50 μl glutathione-agarose beads, with or without purified GFP-LRR (90 μg), for 1 h at 4 °C with end-over-end mixing. The beads were

pelleted by centrifugation for 1 min at 1000 × g at 4 °C, washed 3 times by resuspension and pelleting. After the final wash, the supernatants were discarded, proteins bound to beads were eluted by adding 100 µl of 20mM reduced glutathione in the reaction buffer. Proteins were separated by SDS-PAGE and probed by respective antibodies during Western blotting.

Releasing of NTF by Wnt9B. A sPC1 expression construct consisting of 2 HA tags inserted between the κIgG leading sequence and the 24th amino acid of PC1 was made. Oocytes were injected with the RNA of PC1 and PC2 and cultured for 5 days. The cells were randomly divided into two groups, with one group treated with 300 nM Wnt9B and the other treated with vehicle buffer. After culturing for ~ 16 hours, the culture medium (~1 ml) was collected and concentrated to 100 µl using an Amicon Ultra centrifugal filter (Ultracel-30K from Millipore, UFC803024), and then used for western blotting to detect the released PC1 fragment using an anti-HA antibody. The gel was also stained with Coomassie Blue to detect the GFP-tagged Wnt9B at ~61 kDa. The ~51 kDa band was excised and subjected for amino acid determination after trypsin and chymotrypsin digestion by the University of Iowa Peptide Sequencing Core.

A

Reversal potential (E_{rev})

Constructs \ Cation	PC2	sPC1/PC2	sCTF/PC2	sPC1$_{\text{-RR}}$/PC2	sCTF.Δstalk/PC2
100 Na	-14.13±1.79	-10.99±1.39	-11.60±0.80	-11.2±0.50	-12.61±0.95
100 TEA	-30.84±1.53	-22.69±1.53	-28.60±1.22	-27.23±1.05	-32.29±2.59
100 NMDG	-39.88±1.66	-37.54±1.91	-32.48±2.02	-30.63±0.82	-35.62±2.28
n	5	6	8	8	6

B

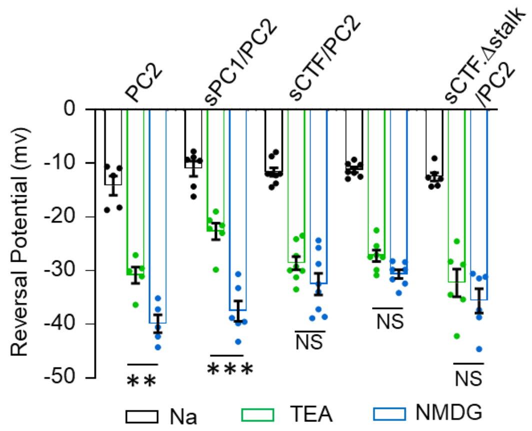

C

Relative permeability

Constructs \ permeability	PC2	sPC1/PC2	sCTF/PC2	sPC1$_{\text{-RR}}$/PC2	sCTF.Δstalk/PC2
P_{TEA}/P_{Na}	0.16±0.01	0.19±0.02	0.14±0.01*	0.15±0.01	0.14±0.01*
P_{NMDG}/P_{Na}	0.11±0.02	0.11±0.01	0.13±0.01	0.14±0.00*	0.12±0.01
P_{TEA}/P_{NMDG}	1.47±0.02	1.84±0.01	1.18±0.01**	1.17±0.00**	1.17±0.01**
n	5	6	8	8	6

*, **; P<0.05, <0.01 vs sPC1/PC2, respectively, by unpaired students t-test for all

D

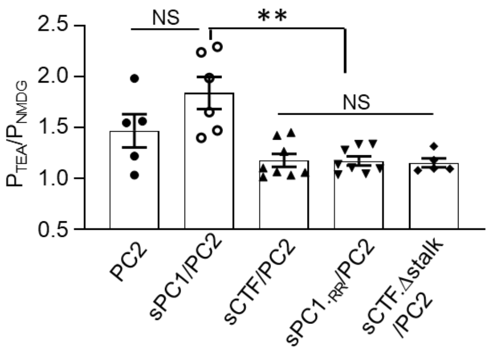

Supplementary Figure 1. Effect of wildtype and mutant PC1 on the permeability to large molecule. (**A**) Oocytes injected with mRNA for PC2 with or without sPC1 or mutants were incubated 100 mM NaCl, tetraethylammonium (TEA)-Cl or N-Methyl-D-glucamine (NMDG)-Cl. Reversal potentials (E_{rev}) were measured as described in the main text Figure 2. (**B**) Open, stippled, and hatched bars are Mean ± SEM of E_{rev} at NaCl, TEA-Cl, and NMDG-Cl, respectively. **, ***; P<0.01, <0.001 as between indicated. (**C**) Relative permeability P_X/P_Y was calculated from measured E_{rev}. *, **; P<0.05, <0.01 vs sPC1/PC2, respectively. (**D**) Mean ± SEM of P_{TEA}/P_{NMDG} from panel C. All statistical analysis by unpaired students t-test. All experiments were repeated two or more times with similar results. Two-tailed unpaired Student's t-test for A, B, C and D.

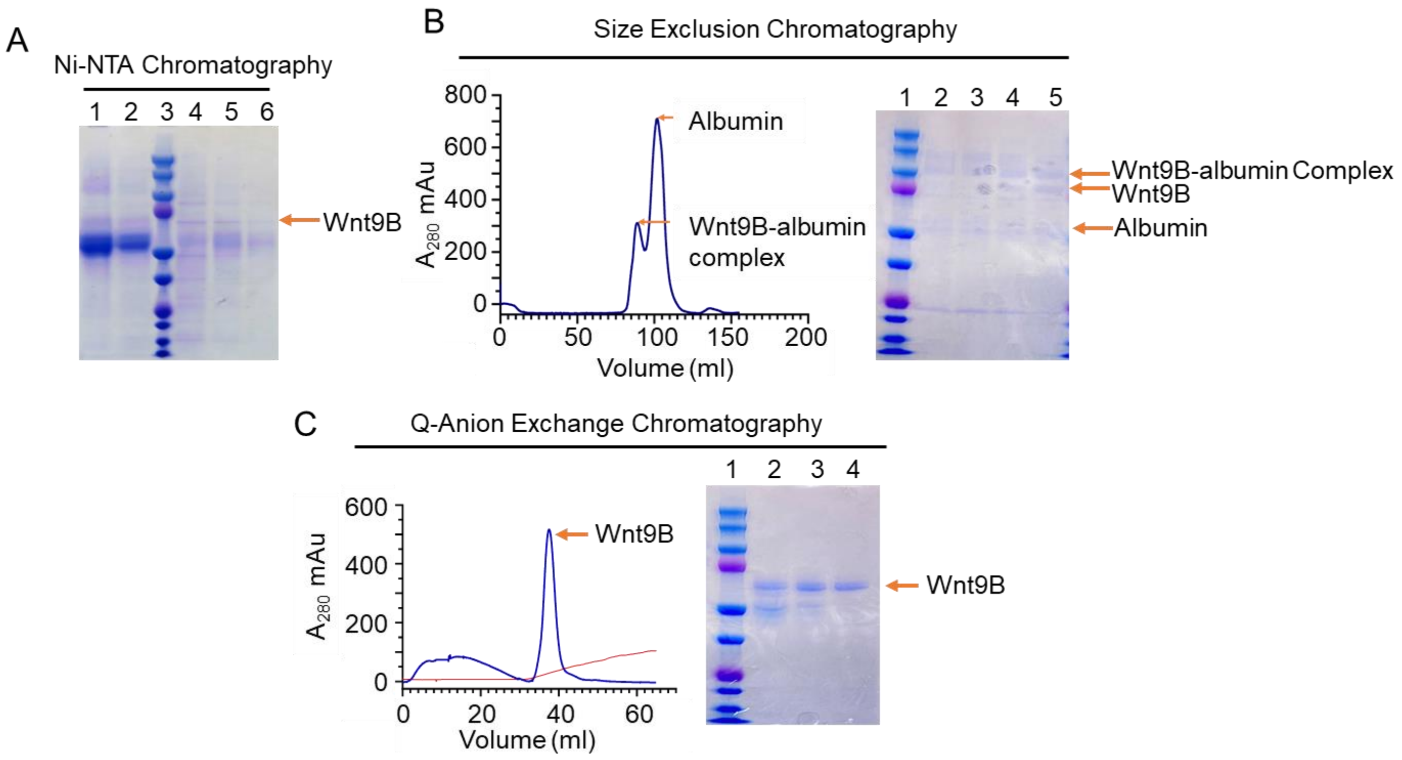

Supplementary Figure 2. Purification of Wnt9B. (**A**) Ni-NTA affinity purification of GFP-tagged Wnt9B from culture supernatants. Coomassie-stained gels showing proteins at different purification steps. Lane 1, flow through; lane 2, wash with 20 mM imidazole; lane 3, molecular weight marker; lanes 4-6, elution of Wnt9B protein with 300 mM imidazole. Red arrow indicates bands corresponding to Wnt9B-albumin complexes. The more abundant bands below correspond to albumin. (**B**) Chromatogram showing Wnt9B protein elution by size exclusion chromatography: the first peak at 76 ml corresponds to Wnt9B-albumin protein; the second peak at 100 ml represents albumin alone. Coomassie-stained gels: lane 1, molecular weight marker, lanes 2-5, fractions corresponding to the first peak eluted at 76 ml. (**C**) Chromatogram of Q-anion exchange chromatography showing elution by increasing NaCl concentration. The peaks eluted at 34-38 ml correspond to Wnt9B protein. Coomassie-stained gels: lane 1, molecular weight marker; lanes 2-4, fractions of elution of Wnt9B protein corresponding to the peaks eluted at 350 mM NaCl.

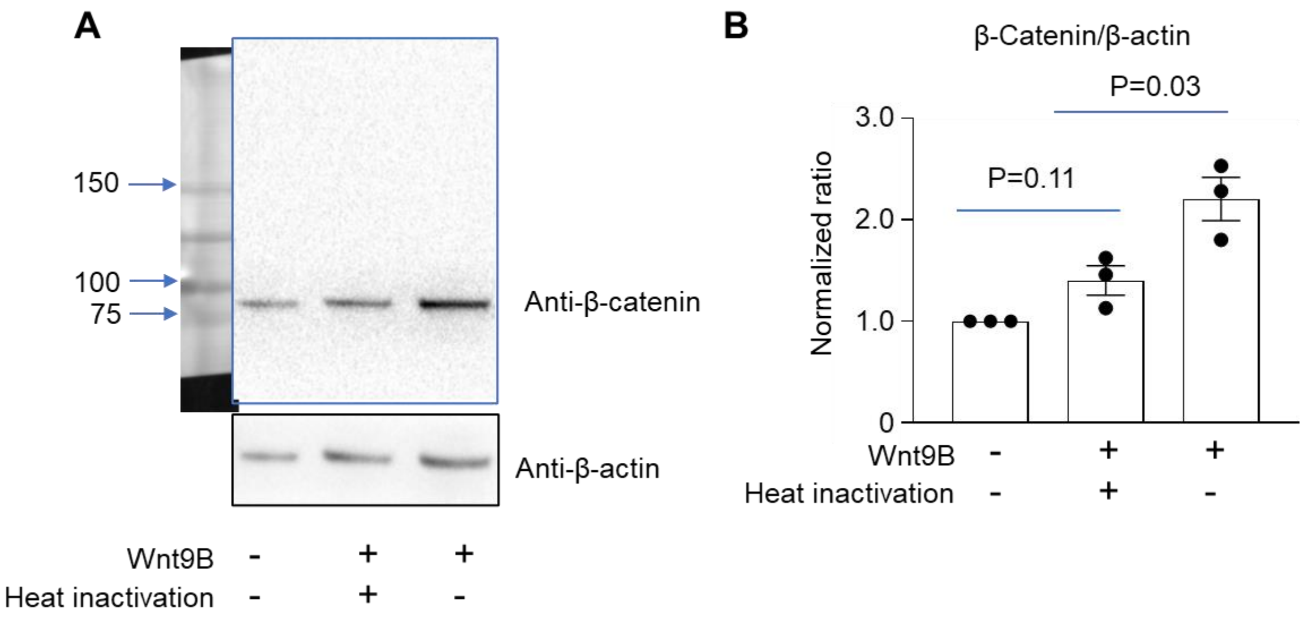

Supplementary Figure 3. Validation of Wnt9B activity. (**A**) HEK293T cells were treated for 16 hours with 300 nM of either freshly prepared or heat-inactivated Wnt9B. Western blotting using anti-β-catenin antibody showed increased β-catenin levels in cells treated with freshly prepared Wnt9B, but not in those treated with heat-inactivated Wnt9B. β-actin, detected using anti-β-actin antibody, served as a loading control and showed similar band intensities across all groups. (**B**) Quantification of the β-catenin/β-actin band intensity ratios, averaged from three independent experiments using three different batches of Wnt9B preparations. For each experiment, the β-catenin/β-actin ratios of all groups were normalized to their control group. Two-tailed unpaired Student's t-test for B.

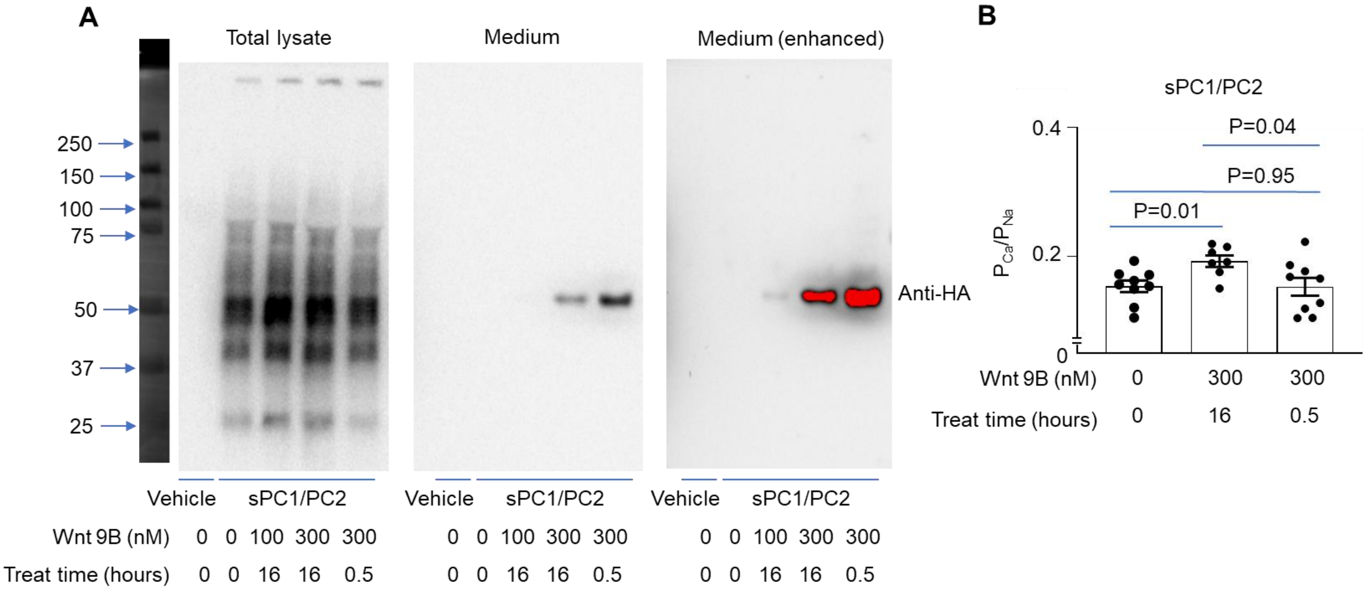

Supplementary Figure 4. Dose- and time-dependent accumulation of the ~51 kDa fragment released by Wnt9B, and the Wnt9B induced changes in Ca^{2+} permeability over time. Oocytes expressing $sPC1^{N.2HA}/PC2$ were treated with Wnt9B at 100 or 300 nM for 16 hours, or with 300 nM for 0.5 hours. Cells treated with protein-dissolving buffer served as controls. (**A**) Western blotting using anti-HA antibody showed that total lysates from all groups exhibited similar levels of sPC1 expression (left panel). In the concentrated culture medium, a ~51 kDa NTF fragment band was detected, with a much stronger signal observed in the group treated with 300 nM Wnt9B for 0.5 hours compared to the 16-hour treatment (middle panel). An enhanced exposure (right panel) revealed a faint ~51 kDa band in the group treated with 100 nM Wnt9B for 16 hours. (**B**) The calculated relative permeability (P_{Ca}/P_{Na}) showed increased Ca^{2+} vs Na^{+} permeability in the group treated with 300 nM Wnt9B for 16 hours, but not in the 0.5-hour treatment group (n=7 to 9 cells each group). Two-tailed unpaired students t-test for B.

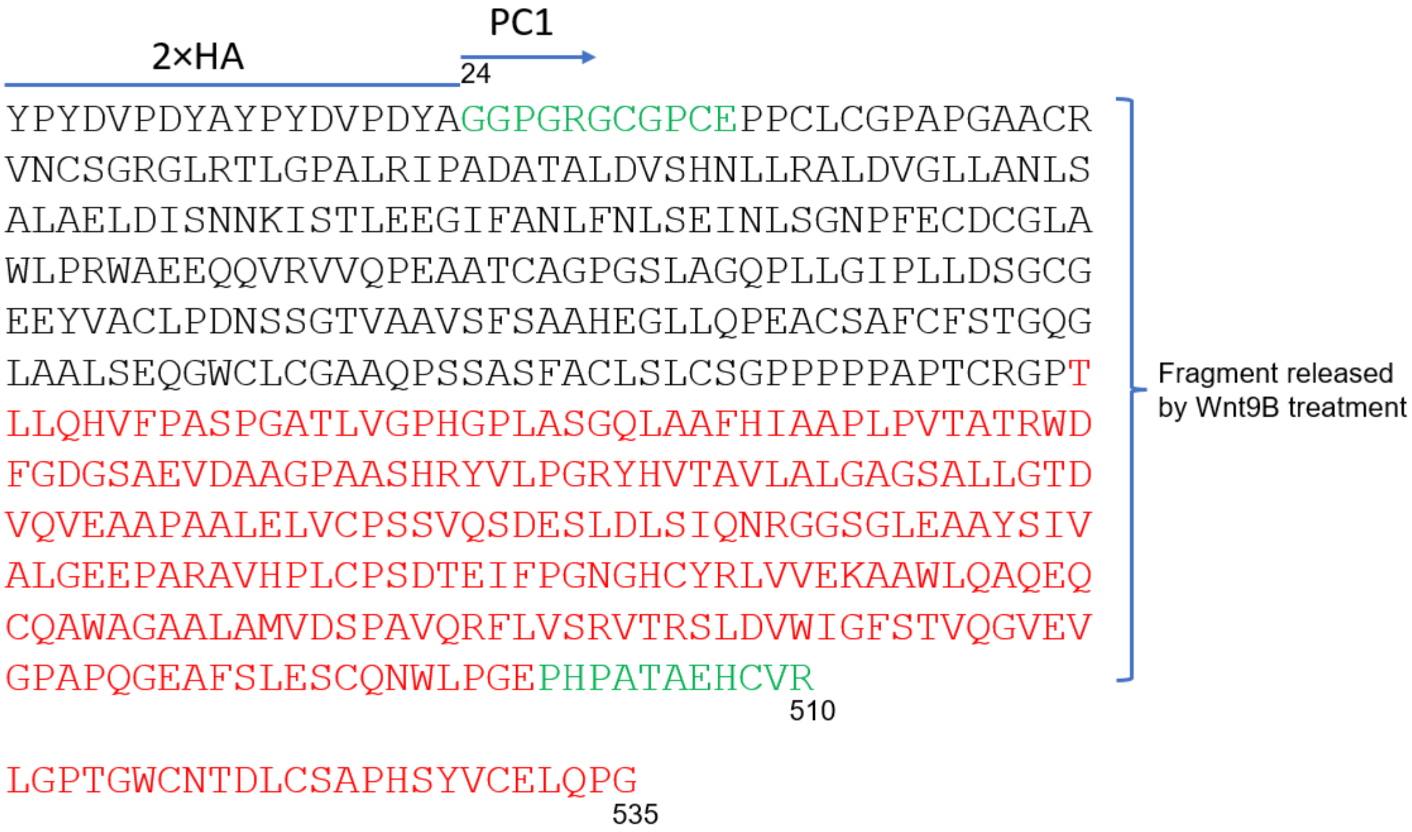

Supplementary Figure 5. Peptide sequencing of the ~51 kDa fragment released by Wnt9B into the medium. The PC1 construct used in this experiment contains 2 HA-tag repeats inserted between the κIgG signal peptide (removed after translation) and amino acid-24 of PC1. Amino acids in red (263-535) are the C-type lectin (CTL) domain. Amino acids labelled between green are revealed by peptide sequencing. Western blotting by anti-HA antibody and peptide sequencing reveal that the fragment released contain HA tag and the majority of CTL domain (up to amino acid 510).

A Reversal potential (E_{rev})

Constructs / Cation	PC2	sPC1/PC2	sCTF/PC2	sCTF.Δstalk /PC2	sCTF. ΔCTT187 /PC2
100NaCl	-11.32±1.42	-10.83±0.68	-11.62±0.67	-11.96±0.15	-11.65±1.06
100KCl	-3.67±1.23	-1.75±0.50	-4.32±0.80	-1.33±0.61 $	-4.25±1.14
100CaCl2	-35.89±1.45	-35.33±1.25	-30.88±1.37 &	-35.93±2.76	-35.53±1.80
100NMDG-Cl	-50.95±3.00	-53.19±3.76	-46.02±2.13	-51.49±8.01	-45.59±4.60
n	7	8	8	4	6

B Relative permeability

Constructs / Cation	PC2	sPC1/PC2	sCTF/PC2	sCTF.Δstalk /PC2	sCTF. ΔCTT187 /PC2
P_K/P_{Na}	1.24±0.01	1.34±0.04 @	1.22±0.02 *	1.39±0.03 #	1.23±0.02
P_{Ca}/P_{Na}	0.15±0.01	0.15±0.01	0.19±0.01 *	0.16±0.01 #	0.16±0.01
P_{Ca}/P_K	0.12±0.01	0.12±0.01	0.15±0.01 *	0.11±0.01 #	0.13±0.01 $
P_{NMDA}/P_{Na}	0.07±0.01	0.06±0.01	0.07±0.02	0.08±0.01	0.09±0.02
n	7	8	8	4	6

Supplementary Table 1. Reversal potentials and calculated relative permeability for oocytes expressing PC2 alone or with sPC1 or deletion mutant constructs. Experiments were performed as described in the main Figure 4. Currents were recorded in bath solution containing (in mM) 100 NaCl, 100 KCl, 100 $CaCl_2$ or 100 NMDG-Cl. (**A**) The reversal potential (E_{rev}) for each cation was determined from I-V curves shown in the main Figure 4. &, $p< 0.05$ for PC2/sCTF vs PC2/sPC1; $, $p< 0.01$ for PC2/sCTF.ΔSTALK vs PC2/sCTF. (**B**) Relative permeability (P_x/P_y) was calculated by measuring potential shifts upon switching extracellular solution. @, $p<0.05$ for PC2/sPC1 vs PC2; *, $p<0.01$ for PC2/sCTF vs PC2/sPC1; #, $p<0.01$ for PC2/sCTF.ΔSTALK vs PC2/sCTF; $, $p<0.05$ for PC2/sCTF.ΔCTT187 vs PC2/sCTF. Two-tailed unpaired students *t*-test for A and B.

A

Reversal potential (E_{rev})

Cation \ Constructs	PC2/sCTF	PC2/sCTF D3072R	PC2/sCTF R3891D	PC2/sCTF D3072R. R3891D	PC2/sCTF R3848E	PC2/sCTF E4078R	PC2/sCTF R3848R. E4078R
100 NaCl	-12.87±0.83	-16.75±0.65	-12.96±2.56	-12.32±1.42	-11.95±0.66	-13.17±0.91	-9.98±2.06
100 KCl	-3.38±1.16	-8.48±1.39	-7.67±2.77	-5.86±1.42	-3.48±0.78	-5.05±1.16	-4.15±2.11
100 CaCl2	-33.79±0.98	-34.13±1.78	-33.02±4.72	-28.56±1.02	-30.83±1.78	-32.13±1.34	-29.64±4.18
100 NMDG-Cl	-41.51±2.91	-34.56±0.41	-27.26±3.99	-33.09±2.18	-34.02±2.22	-34.70±2.62	-26.15±2.65
n	11	4	5	6	8	10	5

B

Relative permeability

Permeability \ Constructs	PC2/sCTF	PC2/sCTF D3072R	PC2/sCTF R3891D	PC2/sCTF D3072R. R3891D	PC2/sCTF R3848E	PC2/sCTF E4078R	PC2/sCTF R3848R. E4078R
P_K/P_{Na}	1.00±0.19	0.96±0.07	** 0.77±0.05	0.82±0.12	0.97±0.12	0.96±0.14	** 0.87±0.12
P_{Ca}/P_{Na}	0.18±0.00	* 0.20±0.02	0.18±0.01	** 0.21±0.01	0.19±0.01	0.19±0.01	0.19±0.03
P_{Ca}/P_K	0.11±0.00	* 0.13±0.00	** 0.15±0.02	*** 0.16±0.02	0.12±0.01	0.12±0.01	0.13±0.02
P_{NMDG}/P_{Na}	0.08±0.01	0.15±0.01	** 0.17±0.01	0.14±0.02	0.13±0.01	0.14±0.01	* 0.16±0.02
n	11	4	6	5	8	10	5

Supplementary Table 2. Reversal potentials and calculated relative permeability for oocytes expressing PC2 with sCTF or mutants. Experiments were performed with the same method as in Supplementary Table 1. (**A**) The reversal potential (E_{rev}) for each cation was determined from I-V curves. (**B**) Relative permeability (P_x/P_y) was calculated by measuring potential shifts upon switching extracellular solution. * P<0.05, **P<0.01, ***P<0.001 compared to PC2/sCTF. Two-tailed unpaired students *t*-test for A and B.

Supplementary Table 3. Abbreviation used in this study

Abbreviation	Full name
PC1 and PC2	polycystin-1 AND polycystin-2
LRR	leucine-rich repeat
ADPKD	Autosomal dominant polycystic kidney disease
GAIN	G-protein coupled receptor autoproteolysis inducing domain
GPS	GPCR proteolysis site
aGPCRs	adhesion G protein-coupled receptors
NTF and CTF	N-terminal fragment and C-terminal fragment
GOF	Gain-of-function
TricB	Trimeric intracellular cation channel isoform-B
Wnt 9B	Wingless-related integration site 9B
Stalk-TOP-PL	Stalk- tetragonal opening for polycystin-pore loop
I_x	Current (I) for x ion
E_x	Reversal potential (E) of x ion
P_x	Permeability of x ion
CHAPS	(3-[(3-Cholamidopropyl) dimethylammonio]-1-propanesulfonate hydrate
AME	anomalous mole fraction effect
NFAT	nuclear-factor-of-activated T-cells
LNAA-PC2	PC2 double mutation leucine-677 and arginine-681 residues to alanine

Supplementary Table 4. Key resources.

Reagent type (species) or resource	Designation	Source or reference	Identifiers	Additional information
Gene (*Homo sapiens*)	hPKD1	GenBank	NM_000296.4	
Gene (Homo sapiens)	hPKD2	GenBank	NM_000297.3	
Animal	Xenopus laevis	Xenopus l	cat# 4800	
Chemical	Collagenase type 4	Worthington	LS004188	1.5 mg/ml
Chemical	Trypsin inhibitor	Worthington	LS003087	0.75 mg/ml
Chemical	T16Ainh-A01	Sigma-Aldrich	SML0493	10 μM
Chemical	BAPTA-AM	Invitrogen	B1205	1-100 μM
Antibody (Rabit)	Anti-HA	Abcam	ab9110	1:500
Antibody (Rabit)	Anti-β actin	ThermoScientific	PA1-16889	1:10000
Antibody (Rabit)	Anti-β catenin	Cell Signaling	cat# 8480	1:1000
Antibody (mouse)	Anti-PC2	Santa Cruz	SC-28331	1:1000
Antibody (goat)	goat anti-mouse IgG-HRP	Invitrogen	31430	1:10000
Antibody (goat)	goat anti-rabit IgG-HRP	SouthernBiotech	4030-05	1:10000
Antibody (goat)	goat anti–rabbit Alexa Fluor 568	Invitrogen	A21069	1:500
RNA synthesis kit	Hiscrib T7 ARCA mRNA kit	New England Biolabs	E2060S	
RNA purification kit	Rneasy mini kit	Qiagen	74104	
DNA mutagenesis	Q5 Site-Directed Mutagenesis Kit	New England Biolabs	E0554S	
DNA mutagenesis	QuickChange II XL Site-Directed Mutagenesis kit	Agilent Technologies,	200521-S	
Software, algorithm	PRISM10	Graphpad		
Software, algorithm	pCLAMP	Molecular Devices		
Software, algorithm	Clampfit11.2	Molecular Devices		

Supplementary Table 5. Constructs used in this study

Construct name	Protein tag	Mutation
PC2	PC2 containing Myc tag at the C-terminus	
HA-PC2	HA tag at the C-terminal end of PC2	
sPC1/PC2	k-IgG signal peptide and Flag tag at N-terminal end and 3×HA tag at C-terminal end of PC1, Myc tag at the C-terminal end of PC2	Deletion of first 23 amino acids signal peptide of PC1
$PC2^{302HA}$	Amino acids 302-306 of PC2 were replaced with HA tag	
$sPC1/PC2^{302HA}$	k-IgG signal peptide and Flag tag at N-terminal end of PC1, HA tag to replace amino acid 302-306 of PC2	Deletion of first 23 amino acids signal peptide of PC1
$F604P^{302HA}$	Amino acids 302-306 of PC2 were replaced with HA tag	F604P in PC2, gain of function mutation
$sPC1/F604P^{302HA}$	k-IgG signal peptide and Flag tag at N-terminal end of PC1, HA tag to replace amino acid 302-306 of PC2	F604P in PC2, deletion of first 23 amino acids in PC1
$PC2_{F604P}$	HA tag at C terminal end of PC2	F604P in PC2
$PC2_{D643K}$	HA tag at C terminal end of PC2	D643K in PC2
$sPC1/PC2_{F604P}$	k-IgG signal peptide and Flag tag at N-terminal end of PC1, HA tag at the C-terminal end of PC2	F604P in PC2
sPC1/PC2	k-IgG signal peptide and Flag tag at N-terminal end of PC1, HA tag at the C-terminal end of PC2	Deletion of first 23 amino acids in PC1
$sPC1_{RR/}PC2$	k-IgG signal peptide and Flag tag at N-terminal end of PC1, 3×HA tag at the C-terminal end of PC1, HA tag at the C-terminal end of PC2	R4100E and R4107E mutations in sPC1
sCTF	kIgG signal peptide and Flag tag at N-terminal end PC1, 3×HA tag at the C-terminal end of PC1	Deletion of first 3048 amino acids in PC1
sCTF.Δstalk	k-IgG signal peptide and Flag tag at N-terminal end PC1, 3×HA tag at the C-terminal end of PC1	Deletion of first 3069 amino acids in PC1
sCTF.ΔCTT	k-IgG signal peptide and Flag tag at N-terminal end PC1, 3×HA tag at the C-terminal end of PC1	Deletion of first 3048 and last 186 amino acids in PC1
sPC1.T3049V/PC2	k-IgG signal peptide and Flag tag at N-terminal end PC1, 3×HA tag at the C-terminal end of PC1, HA tag at the C-terminal end of PC2	Deletion of first 23 amino acids and T3049V in PC1
sCTF.Δstalk. ΔCTT/PC2	k-IgG signal peptide and Flag tag at N-terminal end PC1, 3×HA tag at the C-terminal end of PC1, HA tag at the C-terminal end of PC2	Deletion of first 3069 AND LAST 186 amino acids in PC1
$sPC1^{N.2HA}$	k-IgG signal peptide and 2×HA tag at N-terminal end PC1	Deletion of first 23 amino acids in PC1

Un-cut gel for Figure 1G

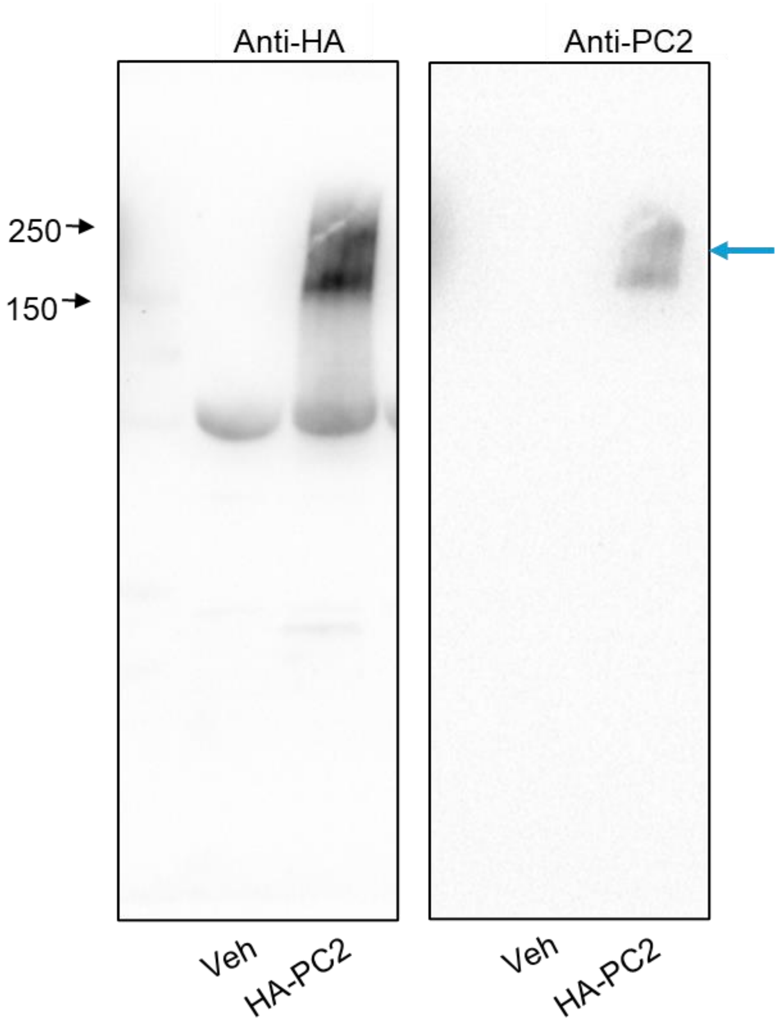

Un-cut gel for Figure 2C

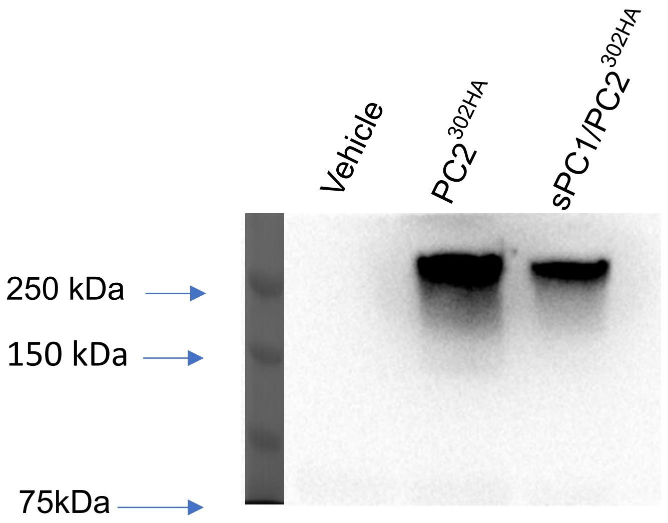

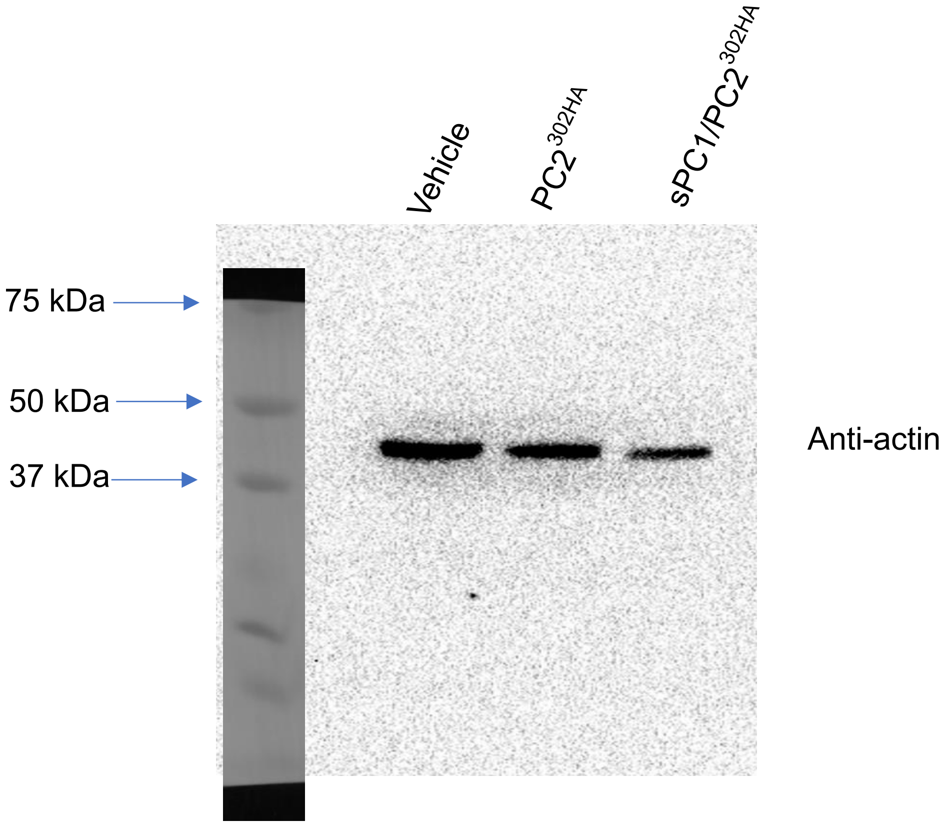

Un-cut gel for Figure 6E

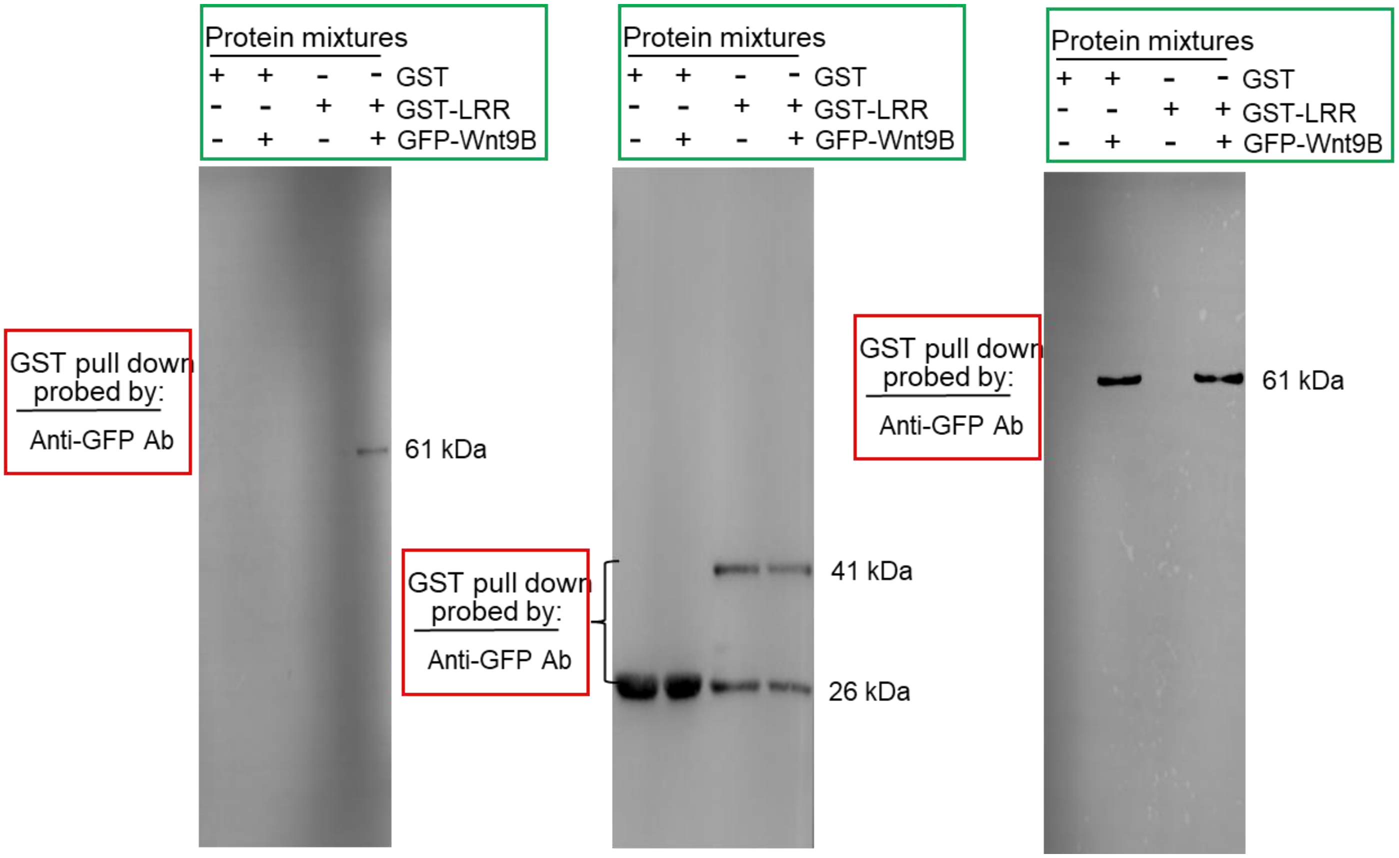

References for supplementry file

1. X. Liu *et al.*, Polycystin-2 is an essential ion channel subunit in the primary cilium of the renal collecting duct epithelium. *Elife* **7** (2018).
2. B. Ng, P. H. Barry, The measurement of ionic conductivities and mobilities of certain less common organic ions needed for junction potential corrections in electrophysiology. *J Neurosci Methods* **56**, 37-41 (1995).
3. E. Neher, Correction for liquid junction potentials in patch clamp experiments. *Methods Enzymol* **207**, 123-131 (1992).
4. N. Tuysuz *et al.*, Lipid-mediated Wnt protein stabilization enables serum-free culture of human organ stem cells. *Nat Commun* **8**, 14578 (2017).
5. E. Mihara *et al.*, Active and water-soluble form of lipidated Wnt protein is maintained by a serum glycoprotein afamin/alpha-albumin. *Elife* **5** (2016).
