# Supplementary material for: Cleavage of N-terminus of polycystin-1 increases calcium permeability of polycystin-1/2 receptor channel complexes
Source: JCI Insight. 2025 Sep 2;10(19):e185186. doi: 10.1172/jci.insight.185186 (PMC12513491; doi:10.1172/jci.insight.185186)

Full unedited gel for:

Cleavage of N-terminus of polycystin-1 increases calcium permeability of polycystin-1/2 receptor channel complexes

Runping Wang, Danish Idrees, Mohammad Amir, Biswajit Padhy, Jian Xie,

and Chou-Long Huang

Department of Internal Medicine, Division of Nephrology, University of Iowa Carver College of Medicine, Iowa City, Iowa, USA

Full unedited gel for Figure 1G

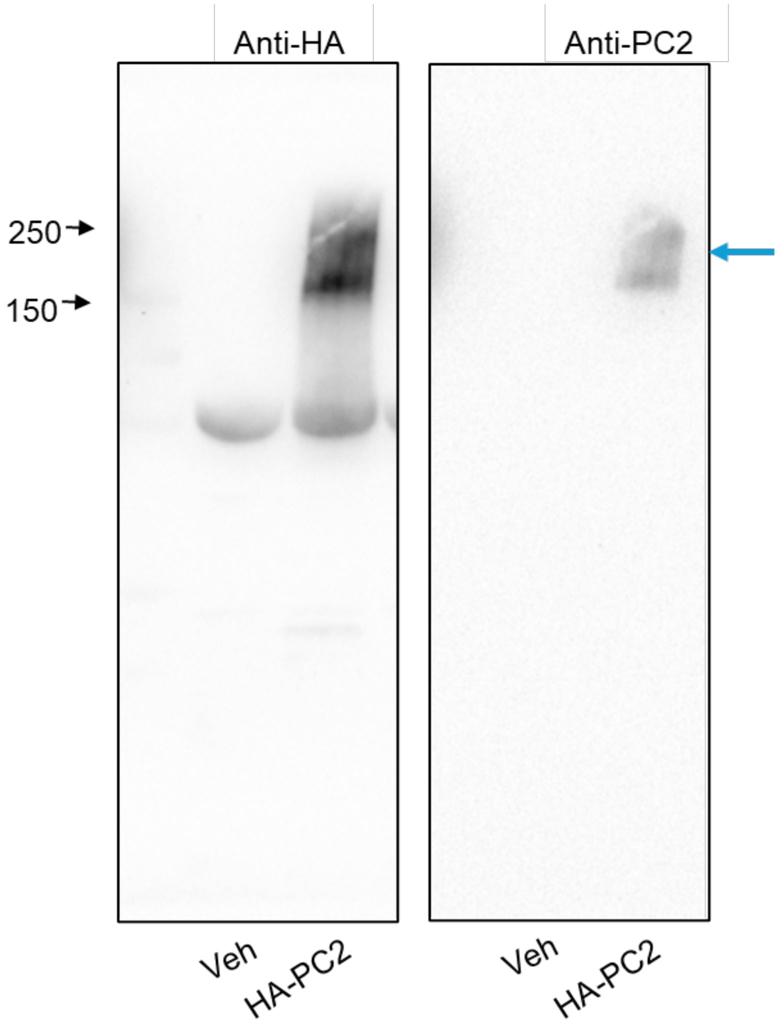

Full unedited gel for Figure 2C

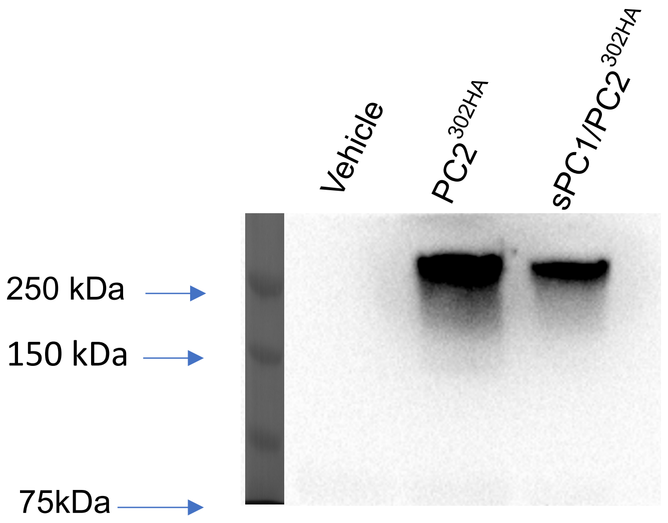

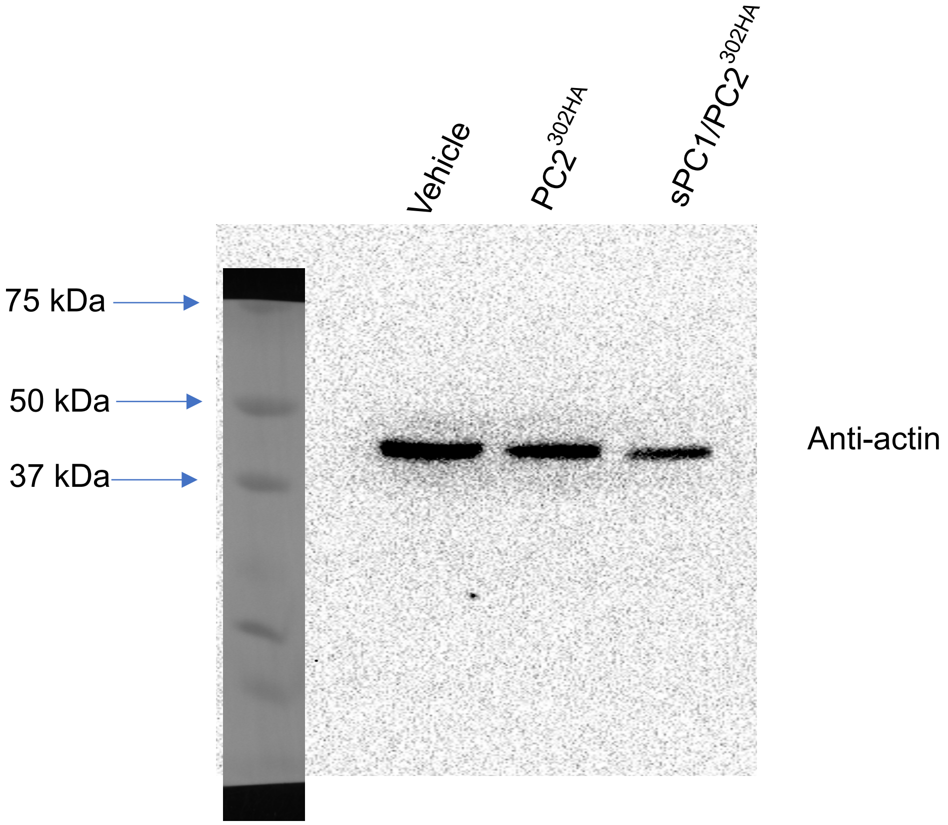

Full unedited gel for Figure 6E

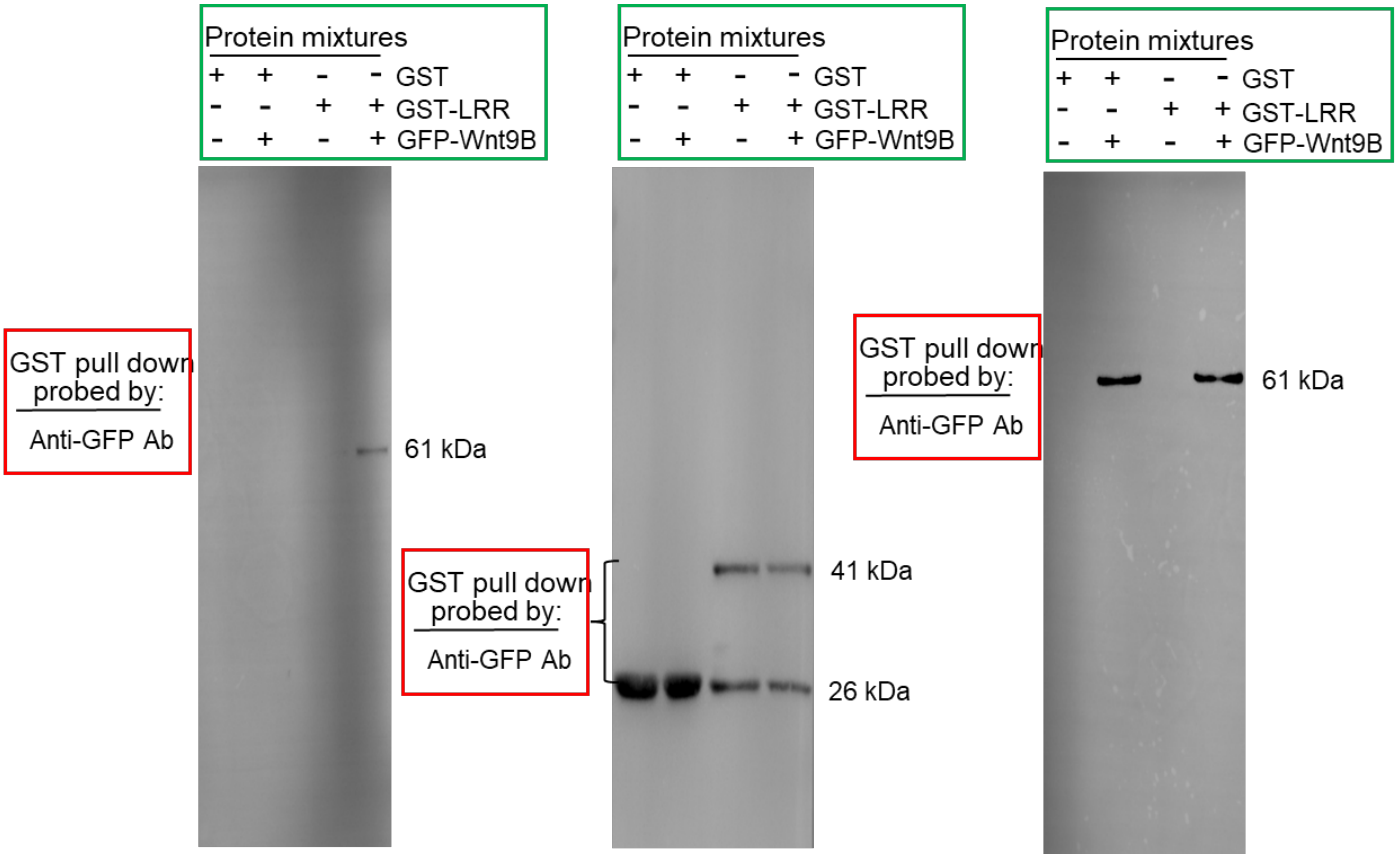

Supplement: Unedited blot and gel images [file jciinsight-10-185186-s222.pdf]
